# The Effects of Dance-Based Exergaming on Mental Rotation, General Motor Coordination, and Math Achievement in Adolescent Students: Nonrandomized Controlled Pilot Study

**DOI:** 10.2196/82610

**Published:** 2026-03-19

**Authors:** Patrick Fargier, Valérian Cece, Vanessa Lentillon-Kaestner, Cédric Roure

**Affiliations:** 1 University of Teacher Education, State of Vaud (HEP VD) Lausanne, State of Vaud Switzerland; 2 University of Lyon, University Claude Bernard Lyon 1, Inter-University Laboratory on Human Movement Biology (LIBM) Villeurbanne France

**Keywords:** dance-based exergaming, exergaming, general motor coordination, math achievement, mental rotation

## Abstract

**Background:**

Exergaming can promote adolescent health by encouraging repetition of game-related tasks, potentially contributing to academic success by developing motor and cognitive skills. Studies have highlighted the influence of exergaming on motor skill learning but have not clarified its effects on general motor coordination. Other studies suggest that exergames may influence math success, particularly with nonmathematical, dance-based exergames, possibly through mental rotation or general motor coordination training. However, the influence of a single exergaming sequence on these abilities and on math achievement in the same participants has not been studied.

**Objective:**

This study aimed to determine whether nonmathematical dance-based exergaming (DEx) improves mental rotation, general motor coordination, and math achievement in adolescent students.

**Methods:**

An experimental group (EG: 15 girls and 15 boys; mean age 14.0, SD 0.7 years) and a control group (CG: 14 boys and 12 girls; mean age 14.2, SD 0.9 years), with no contraindications to physical activity or special educational needs, participated in this nonrandomized controlled study. EG performed DEx involving varied locomotion and interlimb coordination, while CG performed precision ball-throwing exergaming (TEx), requiring catching and throwing. Only DEx involved mental rotation; neither of the two involved mathematical content. Both consisted of 5 weekly 45-minute sessions, implemented using the Lü platform. A pretest-posttest design compared effects on (1) a mental rotation test, (2) locomotion and sprint tests, and (3) quantity comparison plus simple and complex addition and multiplication tests. Each session led to noninstrumented observation, and sessions 3 and 5 included instrumented tracking of physical activity and situational interest.

**Results:**

Monitoring of the sequences highlighted a significant between-group difference in triggered situational interest in session 5 (multivariate analysis of variance: *F*_1,54_=9.15; *P*=.004; η_p_^2^=0.14). This variable was added as a covariate in the analyses of covariance (ANCOVAs), with generalized linear model approach performed further. ANCOVA results showed an advantage for EG over CG in terms of mental rotation (*F*_1,52_=6.17; *P*=.02; η_p_^2^=0.11), number of correct simple additions (*F*_1,52_=8.26; *P*=.006; η_p_^2^=0.14), and error rate in complex addition (*F*_1,52_=8.40; *P*=.005; η_p_^2^=0.14). No other significant results were found.

**Conclusions:**

This study helps clarify the previously unexplored influence of the same exergaming sequence on mental rotation and math skills by showing the positive influence of DEx on mental rotation and only on calculations involving mental rotation, according to the literature. Further research on exergaming is needed to clarify whether such improvement in calculation is linked to mental rotation training itself and might be amplified by the development of general motor coordination. However, the study encourages consideration of integrating exergames into active learning approaches in schools.

## Introduction

### Problem

The digital revolution belongs to a new techno-economic paradigm that has led to the development of a gaming sector with a commercial and entertainment aim [1]. Since the 1970s, this has led to the creation of different genres of video games played using an audiovisual device to interact with a virtual environment under defined rules, conditions, and complexity levels [2]. Such games became part of popular culture, and researchers gradually saw them as potentially useful for academic learning [3]. Studies reported positive effects of practicing entertainment video games on cognitive functions and abilities that promote school achievement [4,5]. The literature also emphasized that video games that embed a pedagogical aim, thus considered serious games, proved useful for sustaining learning activity over time and promoting learning success [6]. Moreover, manufacturers have developed *active* video games, also known as exergames, for entertainment and educational purposes. Although sharing characteristics with conventional video games, exergames require locomotion and movements of bodily segments, not limited to the use of keyboards, joysticks, or other portable controllers [7]. These video games are thus likely to encourage physical activity, and as a result, to have a significant influence on health but also school achievement [8], which remains to be further examined.

### Review of Relevant Scholarship

From the outset, authors have argued that exergames may influence health by favoring physical training and a positive attitude toward physical activity [9]. Consistently, studies showed that exergaming generally elicits, at least in the short term, positive emotions [10-12] and well-being [13,14] in children and adolescents. Other studies showed that the eventual decline over time in the appeal of a given exergame [15] can be avoided by specific practice modalities [10]. Furthermore, the literature reported that such influence on interest may favor energy expenditure [16]. Accordingly, studies found that exergaming may reduce perceived exertion [17], promote intense efforts [18], and have a positive impact on physical fitness [19,20]. On the other hand, the attractiveness of practicing an exergame might favor the repetition of the tasks to be performed when exergaming, thus learning these tasks, as authors have long considered repetition to be a key element of learning [21,22].

Most studies examined the possible influence of exergaming on motor learning outside the school context, and especially fundamental motor skills learning. These encompass “balance/stability skills” (eg, 1-foot balance), “object control skills” (eg, throwing), and “locomotor skills” (eg, jumping), rooted in physical fitness (eg, coordination), and form a basis for learning and performing different types of physical activity [23-25]. Mastery of these motor skills is thus likely to encourage long-term commitment to physical activity, with all its attendant health benefits [26,27]. Interestingly, reviews and meta-analyses highlighted that exergaming may develop balance and postural stability [23,28]. A meta-analysis also found a positive influence of exergames involving actions, such as catching or kicking, on performance in a test assessing object control skills [25]. On the other hand, although a few experimental results suggested the possible influence of exergaming on locomotor skills in children and adolescents [29,30], reviews and meta-analyses found that such findings require confirmation [23]. Especially, whether exergaming might develop general motor coordination (ie, the ability to coordinate several limbs and the whole body [31] as a basis for locomotor skills [23]) remains, to our knowledge, to be clarified.

Furthermore, the influence of exergaming on school achievement has been little studied to date, although studies emphasized that active learning, which links movement and learning, may be a promising way to reduce sedentary behavior while improving academic performance [32,33]. Two recent studies reported a positive influence of practicing exergames with math content on motivation and learning in the areas involved by the exergames, which is in line with the literature on serious games [6]. One of these studies found a positive influence on motivation in math, but not on math achievement, and on physical literacy level [34]. The other study found that exergaming, based on ball throws to hit points after identifying them in a Cartesian coordinate system, increased the performance of a paper-and-pencil task of identifying points in such a coordinate system, as well as the accuracy of ball throws at a target [35]. A 2013 study also reported the influence of practicing a nonmathematical exergame, based on aerobic dance, not only on children’s performance in a 1-mile run, but also on math scores on the Utah Criterion-Referenced Test [36]. The last result thus suggests that practicing an exergame might positively influence school achievement beyond the areas that this exergame targets, which requires further investigation, as such a possibility would be of great pedagogical interest.

This result might be due to the influence of exergaming on executive functions reported in the literature [13,37], as these functions include working memory, attentional flexibility, inhibitory control, and higher-level functions such as reasoning, planning, and problem-solving [38]. However, the explanation seems insufficient, as meta-analyses found that training a cognitive function such as working memory generally leads to progress only in tasks similar to those used in the training [39,40]. Another explanation might lie in the development, thanks to exergaming, of spatial ability, that is, a component of intellectual ability [41] and a predictor of math achievement [42]. A meta-analysis found a correlation between video game skill and spatial ability, but no training influence of video game playing on spatial ability [43]. However, another meta-analysis found that spatial ability may be improved by training, with possible transfer effects [44]. Interestingly, studies found that the practice of physical activities in which the processing of spatial information is decisive (eg, wrestling [45] and dance [46]) may improve mental rotation, that is, a component of spatial ability that allows one to rotate the mental image of an object [47,48]. Other studies showed that mental rotation training may positively influence math achievement [49]. More results suggested the existence of links among mental rotation, general motor coordination, and math achievement, particularly in arithmetic [50-52].

### Hypotheses, Aim, and Objectives

The literature led us to consider that dance-based exergaming (DEx), without explicit math content, could influence mental rotation, general motor coordination, and math achievement. Our study, therefore, aimed to clarify this possible influence in adolescent students.

This study differed from previous studies that examined whether exergames based on math problems and ball-throwing may favor math achievement, physical activity, and ball-throwing accuracy [34,35]. Instead, our study focused on DEx as potentially practiced during physical education (PE) lessons, but also during school periods without formal teaching, or as part of the preparation for a school event. This aimed to examine whether such practice may favor school achievement, as suggested by a previous study that showed a positive influence of exergaming based on aerobic dance on the performance of both an endurance run and a math test [36]. Compared with this study, our study had the distinction of focusing on a dance-based exergame selected as being well-suited to train mental rotation and whose practice was organized to scaffold this training.

The main objective of this study was to determine the influence of this DEx practice on mental rotation efficiency, general motor coordination, and math achievement in an experimental group (EG). This was done by comparison with a control group (CG) involved in precision ball-throwing exergaming (TEx), using a pretest-posttest design. Students’ intensity levels of physical activity during exergaming and the situational interest [53] that this practice generated in them were also measured to monitor their involvement.

## Methods

### Participant Characteristics

This study focused on 2 groups, EG and CG, comprising students aged 13-16 years from a regular secondary school based in Switzerland (Canton of Vaud). A total of 56 students (27 girls and 29 boys) participated in the entire study; EG consisted of 30 students (15 girls and 15 boys) with a mean age of 14.0 (SD 0.7) years, and CG consisted of 26 students (12 girls and 14 boys) with a mean age of 14.2 (SD 0.9) years.

Two qualified PE teachers, with experience in teaching (10 years) and familiarity with their school’s Lü exergaming system, participated in the study (implementation of the exergaming sequences). One was the regular PE teacher for the 2 classes forming EG, and the other for the 2 classes forming CG.

### Inclusion and Exclusion Criteria

We constituted the EG from 2 classes of the school where the experiment took place, and the CG from 2 other classes of this school. On this basis, the inclusion criteria were no known contraindications to physical activity and, for harmonization purposes, no known or presumed special educational needs; thus, not meeting one or more of these criteria led to the a priori exclusion from the study.

### Sampling Procedures

In the enrollment stage (January 2023), 72 students were eligible to participate in the study, 37 as potential EG individuals, and 35 as potential CG individuals. These numbers were likely to decrease depending on consent to participate and then, due to a series of requirements relating to the different phases of the experiment (from February to April 2023): pretests (presence), exergaming (participation in the 5 planned exergaming sessions), and posttests (presence). Among the individuals meeting these criteria, compliance (vs noncompliance) with instructions during testing was the final selection criterion.

### Sample Size, Power, and Precision

Based on preliminary data, a sample size calculation, using the *pwr* package in R software (version 4.5.2; R Foundation for Statistical Computing), indicated that 21 participants per group would be required to achieve 90% power (α=.05, effect size of 0.5) to detect differences in the primary outcomes of the study. Consequently, EG (n=30) and CG (n=26) were deemed acceptable for the study. However, it should be noted that since power calculations were performed for the primary outcomes, other outcomes may have been underpowered.

### Ethical Considerations

The study was conducted in accordance with the Helsinki Declaration of 1975, as revised in 2000. In accordance with local procedure, the project for this pilot interventional study was submitted to the research coordination committee of the host university (University of Teacher Education, Lausanne, Canton of Vaud, Switzerland). This committee approved the project (RCC-pro ID 767) without requiring further submission to the Cantonal Commission for Ethics in Human Research. Subsequently, this project has been integrated into the university’s research project repository.

We also obtained official authorization to implement the study in schools located in the Canton of Vaud and selected 1 secondary school for the study due to its exergaming facilities. We presented the project to the school’s headteacher, who agreed to its implementation. The teachers involved in the experiment, the parents of the students, and the students completed written informed consent forms gathered prior to data collection, which gave them the option to withdraw their consent at any time. No specific compensation was provided for participation in the research. The data were anonymized and stored on a secure university computer. In addition, we have verified that no images that could identify the study participants were used in the study (including in the manuscript and multimedia appendices).

### Design and Conditions

#### Experimental Design

**Figure 1:**
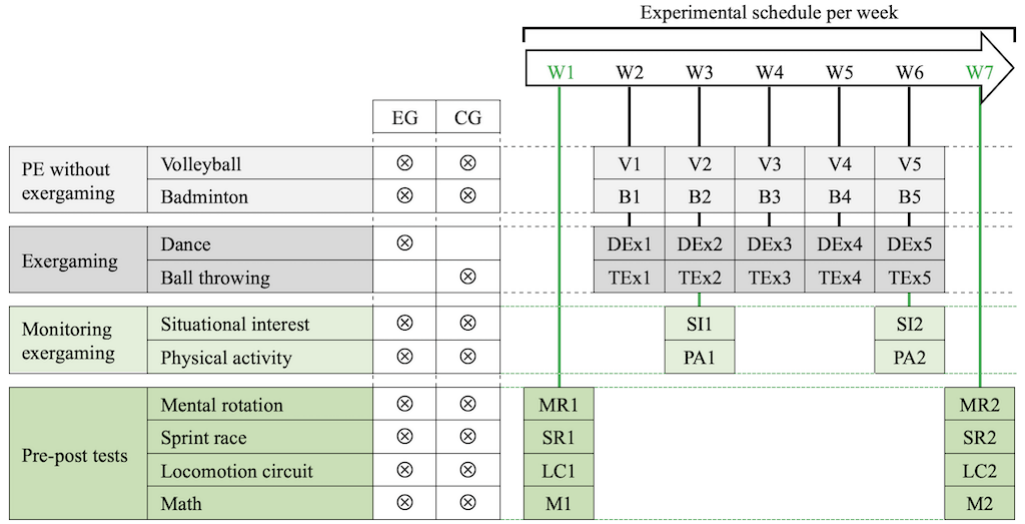
Experimental design to study the influence of a dance-based exergaming sequence, compared with a precision ball-throwing exergaming sequence, on mental rotation, general motor coordination, and math achievement in adolescent students. B1-B5: badminton lessons 1-5; CG: control group; DEx1-DEx5: dance-based exergaming sessions 1-5; EG: experimental group; LC: locomotor circuit, performed during pretests 1 and posttests 2; M1: math tests performed during pretests; M2: math tests performed during posttests; MR1: mental rotation test performed during pretests; MR2: mental rotation test performed during posttests; PA: physical activity monitoring; PE: physical education; SI: situational interest assessments; SR1: sprint race performed during pretests; SR2: sprint race performed during posttest; TEx1-TEx5: precision ball-throwing exergaming sessions 1-5; V1-V5: volleyball lessons 1-5; W1-W7: week 1-7.

The sequences of DEx in EG and TEx in CG were implemented for 5 weeks during the regular PE timetable of the classes involved in the experiment. The participants attended 2 weekly PE lessons: one 90-minute lesson (“2-period lesson”) and one 45-minute lesson (“1-period lesson”). Only the first period of each 2-period lesson differed between EG and CG, with DEx sessions in EG and TEx sessions in CG; each of these weekly exergaming sessions lasted 45 minutes. No student declared previous experience of the exergames practiced during these sequences. Both EG and CG practiced badminton during the second period of each 2-period lesson and volleyball during each 1-period lesson (Figure 1).

The same experimenters observed each session of DEx and TEx to check whether the teacher had implemented it as planned. Furthermore, the intensity levels of the EG and CG participants’ physical activity were measured to check whether these were in line with the respective exergaming demands (Figure 1). This also allowed an estimation of the exergaming contribution to the moderate to vigorous physical activity (MVPA) recommended daily by the World Health Organization [54]. Measurements were done during sessions 3 and 5. The experimenters monitored physical activity, as in previous studies [55], during the sessions, at the end of which situational interest was assessed to control for possible differences between EG and CG likely to have influenced activity during exergaming (Figure 1). These sessions were chosen to avoid novelty bias (session 3) and allow time for possible evolution of situational interest (session 5, ie, the last of DEx and TEx), as in previous studies [35]. Instrumented monitoring of physical activity and situational interest was carried out with each EG and CG participant.

The students completed pretests during the week preceding the beginning of DEx and TEx and posttests during the week after DEx and TEx. During both pretests and posttests, EG and CG performed a mental rotation test, a sprint race, and a locomotion circuit used to assess general motor coordination, and math tests.

#### Exergaming System

**Figure 2:**
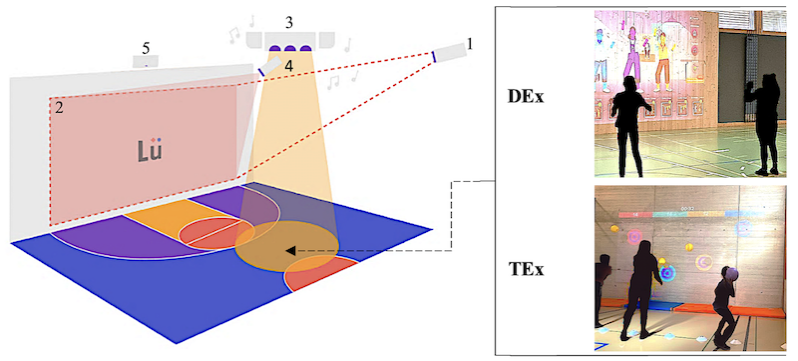
A diagram of the Lü Üno system used to study the influence of dance-based exergaming (DEx), compared with precision ball-throwing exergaming (TEx), on mental rotation, general motor coordination, and math achievement in adolescent students. This system consists of a single-wall configuration with (1) high-definition laser projection (5200 lumens), (2) image (6 m × 3 m), (3) multicolored LED lighting system with moving lights and sound system with dual speakers (2400 Watts), (4) movement detection camera allowing interaction with the projected images, and (5) internal computer with a webcam. The photographs show examples of practice during the experiment with Grööve and Target.

The teachers participating in the study implemented DEx and TEx using a Lü Üno system, that is, an audiovisual infrastructure devoted to exergames practice and coming with gaming applications. In this system, the so-called “heart” module (comprising a PC with a webcam activated by remote control) is connected to an image projection module, a light and sound module, and a camera module for motion detection, for example, to detect the contact of a ball with the wall serving as a projection screen (Figure 2).

#### DEx in EG

**Figure 3:**
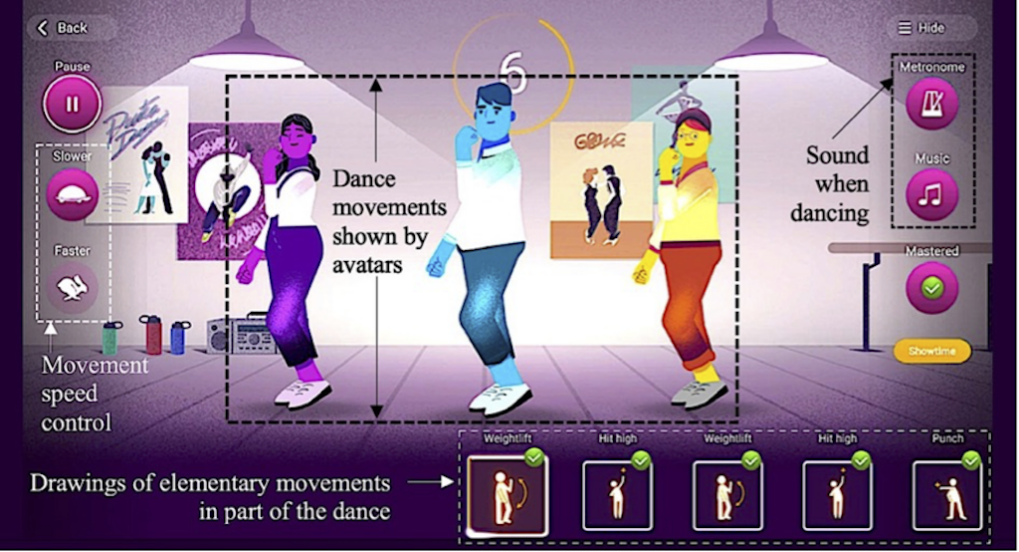
Using the exergame Grööve to implement a dance-based sequence and study its influence on mental rotation, general motor coordination, and math achievement in adolescent students. The screenshot shows an example of movements included in the dance learned during the experiment that were visualizable on a screen. The available options to adapt the practice difficulty to the participants are also shown; these allowed one to control the movements’ speed (x1.00, x0.75, or x0.50) and/or the sound played (metronome only, metronome and music, or music only). The application also allows one to pause the video (“Pause” button) and to save the progress of the participants (“Mastered” button).

During DEx, EG practiced the exergame Grööve (Figure 2) involving no overt math content. Grööve leads to the practice of various dancing steps that drawings indicate, and avatars demonstrate. Grööve also allows one to progressively learn an entire dance using different practice modalities (Figure 3). The choice of this application was in line with the local PE program (“CM32: Develop one’s coordination skills and use one’s own body as a means of expression and communication”).

EG practiced Grööve with the explicit aim of learning a dance based on a song including 4 verses and a chorus. The EG participants had to learn the dance by observing and imitating avatars’ movements (Figures 2 and 3). Sessions 1, 2, and 3 focused respectively on the first and second verses, the refrain, and the third and fourth verses, while sessions 4 and 5 were devoted to reviewing the entire dance (Multimedia Appendix 1). During sessions 1-3, the teacher organized movement learning using part practice [56] and progressively combining the learned parts; during sessions 4-5, the participants reviewed the dance part by part and then entirely, leading to whole practice [56]. Consequently, the content of the sessions varied from one session to another, while ensuring continuity and progressivity in the learning. The teacher also used available options on Grööve (Figure 3) to allow progressive learning from lessons 1-3, as well as repetition under facilitating conditions and in final conditions during lessons 4 and 5, respectively (Multimedia Appendix 1).

Each part of the dance was largely composed of asymmetric movements that avatars showed facing the EG participants; however, the avatars were also turning, so that the EG participants were seeing them in profile (Multimedia Appendix 1). Thus, mental rotation was useful to observe and imitate the avatars’ movements. However, it was possible to imitate correctly part of these movements without using mental rotation. For example, when the participants viewed an avatar from the front, arms uncrossed, they could imitate an arm elevation seen on their left by deciding, without using mental rotation, to elevate their own right arm.

Thus, to maximize the use of mental rotation, the teacher had to instruct the EG participants to imagine themselves dancing in the position of one of the avatars facing them, that is, to rotate their own body mentally to get into this position. The teacher had to give this instruction before each dancing phase, to reiterate it during learning, and when providing feedback. Each session, the teacher also had to film the dance movements of the EG participants, with the Lü camera used facing them (Figure 2). At the end of the session, the teacher had to use the videos for formative evaluation and to train the participants to analyze freeze-frames with the same instruction as when imitating an avatar (eg, to determine which arm of the viewed participant was outstretched).

#### TEx in CG

During TEx, exergaming in CG was based on precision ball-throwing (Figure 2), which was compliant with the local PE program (“CM32: Coordination training [...] using balls”). TEx involved no overt math content and no relevant use of mental rotation.

The teacher implemented TEx using 8 Lü applications, each leading to throwing a ball at a target, in addition to catching it and performing simple walk or run locomotion (Multimedia Appendix 2). These applications were (1) Target (throw a ball at a circular target, with the target location changing automatically as the game progresses), (2) Galactic (throw a ball at asteroids to protect a spaceship), (3) Relé (relay race with a run to reach a floor line, a ball-throw at the team zone on the screen, then a ball catch to throw it to a partner), (4) Spörts (perform basketball shots at a virtual basket), (5) Germ (throw a ball to create obstacles preventing a virus to reach own zone and allowing to direct this virus to the adverse zone), (6) Vïka (throw a ball at targets to answer questions, then propel a boat toward a village, and protect villagers from a monster), (7) Lüvia (puzzle solving: throw a ball at the correct answer displayed on the screen among other possible answers), and (8) Brüsh (throw a ball at candy before it hits an avatar’s teeth, while avoiding the floss heading toward the avatar).

Throughout TEx, the common thread in terms of learning consisted of practicing precision ball-throwing. On this basis, the experimenters and the teacher involved in TEx designed the sessions to ensure a mix of continuity, variety, and progressivity in practice. Each session involved the use of 3 of the applications described above in the following chronological order: Target, Galactic, and Relé during session 1, Target, Galactic, and Spörts during session 2, Germ, Vikä, and Relé during session 3, Lüvia, Brüsh, and Spörts during session 4, and Lüvia, Spörts, and Vikä during session 5. In addition to this mix of novelty and reuse in applications from one session to another, the teacher initially had to adjust the task requirements to suit the participants’ capacities, then gradually increase these requirements, using the options available for each application. For example, the participants had to play Target with targets shown in the same screen column and then scattered across the entire screen (Multimedia Appendix 2).

#### Volleyball and Badminton Sequences

During the experiment, EG and CG separately took part in the same weekly 45-minute badminton and volleyball lessons (Figure 1). Each lesson began with a conventional warm-up (running, stretching, and muscle strengthening), followed by tasks focused on a sport-specific technique, and then regular matches. The content of the lessons was in line with the local PE program (“CM34: Master basic sports techniques, adapt to the game situation”).

### Measures and Covariates, Data Collection, Quality of Measurements

Before the experiment, each sequence was tested with other students than those forming the EG and CG. This enabled the teachers involved in the study to perfect their use of the Lü Üno system, as DEx and TEx included elements (content, practice options) that they needed to explore further. This also led to adjusting the sequences for subsequent implementation.

The experimenters conducted a noninstrumented observation of each of the 5 DEx and TEx sessions implemented (Figure 1) to enable comparison between the observed session and the planned session regarding content, teacher intervention, and participant activity.

For confirmation purposes, during sessions 3 and 5 of DEx and TEx, the experimenters measured the intensity levels of physical activity achieved by each EG and CG participant (Figure 1). The examined variables were (1) the moderate and vigorous intensity levels, expressed in seconds and percentage of time activity, and (2) MVPA, expressed similarly to the moderate and vigorous intensity levels. Given the exergaming characteristics in DEx and TEx described above, we expected EG to be more involved than CG in vigorous-intensity activity, and CG more involved than EG in moderate-intensity activity, as the musical tempo in DEx was to be increased during session 3 and remain at peak in session 5, whereas no such activity peaks were planned in TEx.

Directly after sessions 3 and 5, the experimenters assessed the situational interest of each EG and CG participant to control for possible between-group differences likely to have influenced participants’ activity (Figure 1). The measurement of interest, as conceived in the field of educational psychology [57], is well-suited to examine the closeness and sustainability of a person’s relationship to learning depending on the content to be learned [58]. The triggered situational interest in a person depends on the present situation and personal characteristics [53]. This may lead to maintained situational interest, eventually allowing the gradual stabilization of interest. Interestingly, studies also showed that situational interest may influence physical activity intensity levels and energy expenditure during physical activity [59].

During both pretests and posttests (Figure 1), the EG and CG participants performed a mental rotation test and math tests at the beginning of 2 separate math lessons. On the days these tests were administered, no specific physical activity was performed beforehand (PE classes or otherwise) to promote harmonization of testing conditions. The participants performed a sprint race during the 1-period PE lesson (45 minutes) of the week, and a locomotion circuit during the 2-period PE lesson (90 minutes).

The experimenters also asked science and math teachers in classes of the EG and CG participants not to provide any specific training on mental rotation and the mathematical content covered in the pretests and posttests during the experiment.

### Instrumentation

#### Monitoring Intensity Levels of Physical Activity and Situational Interest

During sessions 3 and 5 of DEx and TEx, the experimenters measured the intensity levels of physical activity (moderate, vigorous, and MVPA) achieved by each EG and CG participant using hip-worn GT3X+ accelerometers (Actigraph LLC), providing a receivable estimation of physical activity in free-living conditions [60]. The accelerometer data were processed using ActiLife6 (Actigraph LLC). This led to converting the data into activity counts that were summed and recorded using a specific time interval known as an “epoch” (selectable duration from 5 seconds to 1 minute). The selected epoch was 5 seconds, as the study was focused on acute and intermittent exercise bouts.

In this study, situational interest was assessed using the French situational interest questionnaire [61] that contains three factors: (1) triggered situational interest (participant’s perception of the game demanding full attention), (2) maintained situational interest feeling (to what extent the activities were found enjoyable), and (3) maintained situational interest value (importance allocated to success during the game). To complete the questionnaire, the participants had to rate statements on a 5-point Likert scale (Multimedia Appendix 3 [53,57,58,61]); for each factor, this led to scores ranging from 1 (lowest possible score) to 5 (highest possible score).

#### Pretests and Posttests

The EG and CG participants completed the French version [62] of Vandenberg and Kuse’s mental rotations test [63] based on a comparison of images of geometrical structures made of 10 cubes (Multimedia Appendix 4 [52,62-70]), the scores ranging from 0 to 40 points, with 40 corresponding to a perfect performance.

These participants also performed a locomotion circuit called the Harre circuit test in the literature [64] to assess general motor coordination [65], with reaction to an audible signal, a forward roll, running phases with direction changes, and phases with jumping over a bench, then crawling under it (Multimedia Appendix 4). In line with previous studies [52,66], a sprint race (20 meters) was performed separately to control for the influence of sprinting capacities on the circuit performance. The performance of the locomotion circuit and of the sprint race was timed using the same timing system (Race Link Racing System; Brower Timing Systems).

Moreover, the participants performed 2 separate paper-and-pencil series of quantity comparisons, based on a previous study [67] (Multimedia Appendix 4), that is, a series of comparisons between 2 sets of points and a series of comparisons between 2 single-digit numbers. The participants also performed 4 separate paper-and-pencil series of mental calculations, based on a previous study [68] (Multimedia Appendix 4), that is, a series of simple additions (2 single-digit numbers), complex additions (three 2-digit numbers), simple multiplication (2 single-digit numbers), and complex multiplication (a 2-digit number and a single-digit number). For each task series listed above, we considered the total number of processed items, the number of correct results, and the error rate.

### Masking

The EG and CG students, as well as the teachers involved in the study, were informed of the main lines of the study but were left unaware of any expected results. DEx and TEx sessions have been designed separately, each by a group including the researchers and the PE teacher who would teach the sequence (either to EG or to CG).

### Data Diagnostic, Psychometrics, and Analytic Strategy

Statistical analyses were performed without missing data, due to sampling procedures (see “Sampling Procedures” subsection above) using IBM SPSS Statistics (version 30.0.0.0; 171). On this basis, the normality of the numerical variables was preliminarily checked, and the internal reliability of the situational interest scales was evaluated using Cronbach α coefficients.

Multivariate analysis of variance (MANOVA) tests were performed to compare the intensity levels of physical activity in EG and CG during sessions 3 and 5 of DEx and TEx. The scores for each intensity level considered were specified as dependent variables and the group condition (EG vs CG) as a fixed factor. Similarly, MANOVA tests were done to examine possible differences between EG and CG in scores for each of the 3 situational interest factors measured after sessions 3 and 5.

For each variable measured at pretest and posttest, analysis of covariance (ANCOVA) tests were performed using a generalized linear model approach [71]. The model included basically the posttest score as the dependent variable, the group (EG vs CG) as a fixed factor, and the pretest score as a covariate; in the event of a between-group difference regarding a situational interest variable, it was planned to add the variable as a covariate. Beyond data normality, variance homogeneity, heteroskedasticity, and regression homogeneity were checked; on such a basis, ANCOVA was computed either on untransformed data or on ranks, using the same method for the computations [71,72].

For all the analyses, partial eta squared (η_p_^2^) provided an index of effect size characterizing the effect as small, medium, or large, depending on whether the value of η_p_^2^ was between 0.01 and 0.06, between 0.07 and 0.14, or >0.14, respectively [73]. Contrast analyses were applied in the case of significant results.

## Results

### Recruitment and Participant Flow

**Figure 4:**
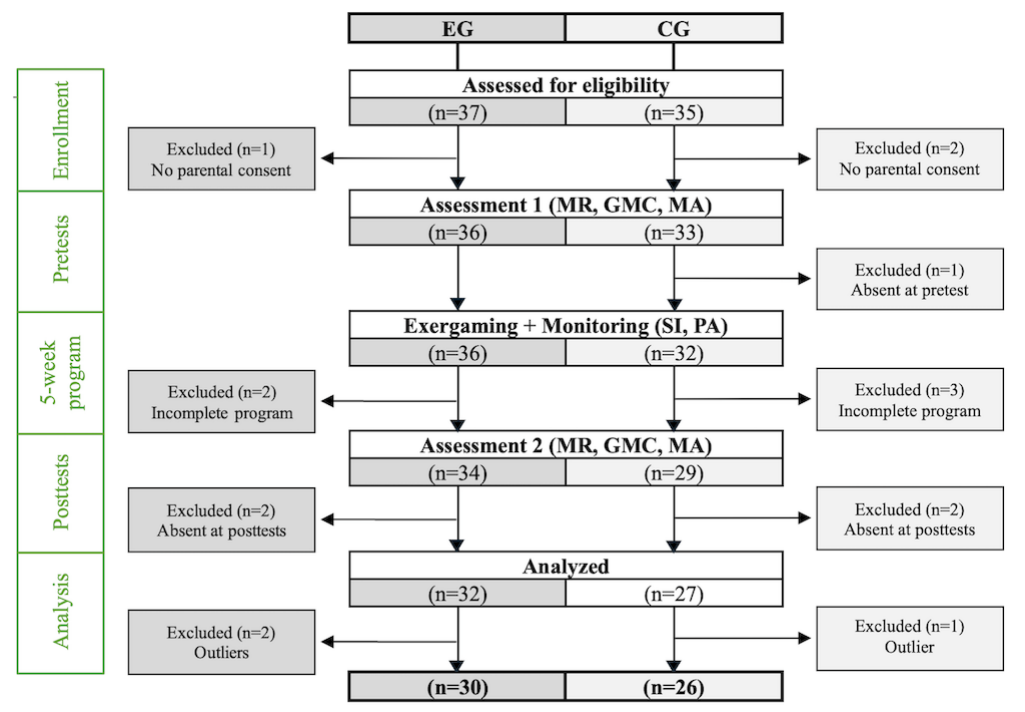
Study flowchart. Steps to form experimental group (EG; dance-based exergaming) and control group (CG; precision ball-throwing-based exergaming) to study the influence of dance-based exergaming, compared to precision ball-throwing exergaming, on mental rotation (MR), general motor coordination (GMC), and math achievement (MA) in adolescent students: enrollment stage (January 2023), pretest, 5-week program, and posttests (from February to April 2023). Three participants, among those having participated in all pretests, posttests, and exergaming sessions, were excluded due to failure to follow instructions during testing (random responses). PA: physical activity intensity levels; SI: situational interest.

The different stages involved in forming the groups, which resulted in the selection of 30 students (15 girls and 15 boys) for EG (mean age 14.0, SD 0.7 years) and 26 students (12 girls and 14 boys) for CG (mean age 14.2, SD 0.9 years), are presented in Figure 4.

### Statistics and Data Analysis

#### Intensity Levels of Physical Activity and Situational Interest

The observation of each DEx and TEx session led to the conclusion that the sessions were taught as planned and that the students completed the exergaming tasks. Such observation was supplemented by the results of analyses of physical activity intensity levels and situational interest during sessions 3 and 5.

The data relating to physical activity intensity levels were normally distributed (skewness values ranging from –0.64 to 0.79, SE 0.32; kurtosis values ranging from –0.58 to 2.56, SE 0.63). For each MANOVA (Table 1), Pillai trace values ranged from *F*_3,52_=10.44 to *F*3,52=63.63 (*P*<.001).

**Table 1:** Results of multivariate analysis of variance (MANOVA) tests for physical activity intensity levels. Physical activity intensity levels were monitored during sessions 3 and 5 of 2 exergaming sequences (5 weekly sessions of 45 minutes each) as part of a study of dance-based exergaming (DEx) influence on mental rotation, general motor coordination, and math achievement in adolescent students. The influence of DEx (experimental group [EG]) was compared to that of precision ball-throwing exergaming (control group [CG]).

			EG (n=30), mean (SD)		CG (n=26), mean (SD)		MANOVA tests							Contrasts
							*F* test (*df*)		*P* value		η_p_^2^			
**Session 3 (seconds)**														
	Moderate	739.7 (122.7)		1060.8 (154.4)		75.10 (1, 54)		<.001		0.58		EG < CG		
	Vigorous	336.7 (82.2)		283.6 (139.4)		3.10 (1, 54)		.08		0.05		N/A^b^		
	MVPA^a^	1180.7 (173.5)		1419.2 (290.3)		14.16 (1, 54)		<.001		0.21		EG < CG		
**Session 3 (%^c^)**														
	Moderate	35.9 (5.3)		42.0 (5.5)		17.75 (1, 54)		<.001		0.25		EG < CG		
	Vigorous	16.4 (4.0)		12.5 (5.9)		8.46 (1, 54)		.005		0.13		EG > CG		
	MVPA	57.4 (8.0)		56.1 (3.3)		0.30 (1, 54)		.59		0.005		N/A		
**Session 5 (seconds)**														
	Moderate	724.7 (102.5)		1035.0 (164.3)		73.94 (1, 54)		<.001		0.58		EG < CG		
	Vigorous	322.7 (63.3)		221.0 (115.3)		17.34 (1, 54)		<.001		0.24		EG > CG		
	MVPA	1189.3 (154.1)		1311.9 (291.7)		4.01 (1, 54)		.05		0.07		N/A		
**Session 5 (%^d^)**														
	Moderate	32.1 (4.4)		40.9 (5.6)		42.01 (1, 54)		<.001		0.44		EG < CG		
	Vigorous	14.3 (2.9)		10.0 (5.5)		14.72 (1, 54)		<.001		0.21		EG > CG		
	MVPA	52.8 (6.9)		51.7 (10.3)		0.17 (1, 54)		.68		0.003		N/A		

^a^MVPA: moderate to vigorous physical activity.

^b^N/A: not applicable.

^c^Intensity levels of physical activity in percentage of the total physical activity time during session 3.

^d^Intensity levels of physical activity in percentage of the total physical activity time during session 5.

On average, during session 3, the EG participants spent 1180.7 (SD 173.5) seconds in MVPA, that is, 57.4% (35420/61770 seconds) of total physical activity time, and the CG participants, 1419.2 (SD 290.3) seconds, that is, 56.1% (36900/64745 seconds) of total physical activity time. During session 5, the values were 1189.3 (SD 154.1) seconds and 52.8% (34110/64765 seconds) in EG, 1311.9 (SD 291.7) seconds and 51.7% (35680/67680 seconds) in CG (Table 1).

CG had more MVPA time than EG in sessions 3 (*P*<.001; large effect size) and 5 (*P*=.05; small effect size), but no between-group difference was found when considering MVPA as a percentage of total physical activity time in sessions 3 (*P*=.59) and 5 (*P*=.68). As expected, MVPA relied on (1) significantly higher moderate-intensity values in CG than in EG, in seconds and percentage of total physical activity time during sessions 3 and 5 (*P*<.001; large effect sizes), and (2) higher vigorous-intensity values in EG than in CG, in seconds and percentage of total physical activity time during sessions 3 and 5, with significant differences (*P*<.001; medium to large effect sizes), except for values in seconds during session 3 (*P*=.08).

On the other hand, data on situational interest in sessions 3 and 5 were normally distributed (skewness values from –0.50 to 0.47, SE 0.32; kurtosis values from –1.19 to –0.83, SE 0.63). In addition, the Cronbach α coefficients were good for each situational interest factor measured after sessions 3 (from 0.89 to 0.92) and 5 (from 0.90 to 0.92). Results of MANOVA tests reached statistical significance for session 5 (Pillai trace with *F*_3,52_=3.93; *P*=.01), but not for session 3 (*F*_3,52_=1.09; *P*=.36), due to a higher score for triggered situational interest in EG than in CG (*P*=.004; large effect size) in session 5 (Table 2). Consequently, triggered situational interest during session 5 was added as a covariate to perform ANCOVA tests in further analyses.

**Table 2:** Results of multivariate analysis of variance (MANOVA) tests for situational interest. Situational interest factors were assessed directly after sessions 3 and 5 of 2 exergaming sequences (5 weekly 45-minute sessions each) as part of a study of dance-based exergaming influence on mental rotation, general motor coordination, and math achievement in adolescent students. The influence of dance-based exergaming (experimental group [EG]) was compared to that of precision ball-throwing-based exergaming (control group [CG]).

		EG (n=30), mean (SD)	CG (n=26), mean (SD)	MANOVA tests				Contrasts
				*F* test (*df*)	*P* value	η_p_^2^		
**Situational interest in session 3**								
	TSI^a^	3.52 (1.39)	2.89 (1.21)	3.23 (1,54)	.08	0.06	N/A^b^	
	MSIF^c^	2.63 (1.17)	2.21 (1.05)	1.98 (1,54)	.16	0.03	N/A	
	MSIV^d^	2.59 (1.13)	2.16 (1.10)	2.04 (1,54)	.16	0.04	N/A	
**Situational interest in session 5**								
	TSI	3.49 (1.23)	2.47 (1.29)	9.15 (1,54)	.004	0.14	EG > CG	
	MSIF	2.34 (1.05)	2.02 (1.12)	1.23 (1,54)	.27	0.02	N/A	
	MSIV	2.42 (1.10)	2.08 (1.14)	1.34 (1,54)	.25	0.02	N/A	

^a^TSI: triggered situational interest.

^b^N/A: not applicable.

^c^MSIF: maintained situational interest feeling.

^d^MSIV: maintained situational interest value.

For all MANOVA results, the estimated marginal means and corresponding 95% CIs are given in Multimedia Appendix 5.

#### Pretests, Posttests, and Exergaming Influence

ANCOVAs were performed on ranks for the total number and the number of correct comparisons of dot ensembles performed (heteroskedasticity test with *F*_1,54_=4.88; *P*=.03 and *F*_1,54_=4.18; *P*=.046), all data series for number comparison (kurtosis values from 2.60 to 4.90, SE 0.63), the total number of calculations in simple addition (Levene’s test with *F*_1,54_=7.94; *P*<.001), error rate in simple (kurtosis values of 3.02 and 14.82, SE 0.63) and complex (Levene’s test with *F*_1,54_=18.72; *P*<.001) additions, and in simple multiplication (kurtosis values of 3.26 and 15.50, SE 0.63). Untransformed data were used in any other case. No significant regression homogeneity issue was found (results ranging from *F*_1,50_=0.003; *P*=.95 to *F*_1,50_=3.05; *P*=.08).

The results showed that EG had a statistically significant advantage (medium to large effect sizes) over CG regarding the Vandenberg and Kuse mental rotations test scores (*P*=.02), the number of correct results of simple additions (*P*=.006), and the error rate in complex additions (*P*=.005). No other significant differences were found, either in terms of additions or in measurements relating to the sprint race, the locomotion circuit, quantity comparisons, or multiplications (Table 3).

**Table 3:** Results of analysis of covariance (ANCOVA) tests. Effects of dance-based exergaming on test scores of mental rotation, general motor coordination, and math in adolescent students. Comparison of test scores obtained after a dance-based exergaming sequence of five 45-minute weekly sessions (experimental group [EG]) with those obtained after a similar sequence based on precision ball-throwing (control group [CG]), controlling for the scores before the first exergaming sessions and the triggered situational interest levels during exergaming.

			Pretests, mean (SD)				Posttests, mean (SD)				ANCOVA tests							Contrast
			EG (n=30)		CG (n=26)		EG (n=30)		CG (n=26)		*F* test (*df*)		*P* value		η_p_^2^			
**VMRT^a^**																		
	Score	18.40 (7.98)		13.77 (7.51)		22.23 (8.95)		13.88 (8.35)		6.17 (1, 52)		.02		0.11		EG > CG		
**GMC^b^**																		
	Sprint	3.60 (.41)		3.62 (.34)		3.65 (.30)		3.63 (.38)		0.18 (1, 52)		.68		0.003		N/A^c^		
	LC^d^	22.41 (4.15)		22.42 (3.44)		21.72 (4.39)		21.20 (3.41)		0.86 (1, 52)		.36		0.02		N/A		
**DC^e^**																		
	N^f^ (r^g^)	59.43 (9.03)		60.81 (7.27)		64.63 (8.76)		67.23 (6.22)		0.66 (1, 52)		.42		0.01		N/A		
	C^h^ (r)	55.20 (5.23)		56.85 (5.26)		58.73 (8.14)		61.27 (4.57)		0.01(1, 52)		.93		0.0001		N/A		
	ER^i^	6.23 (7.03)		6.15 (4.41)		8.92 (7.36)		8.61 (4.24)		0.08 (1, 52)		.77		0.002		N/A		
**NC^j^**																		
	N (r)	65.53 (7.08)		66.15 (6.42)		68.96 (6.10)		68.81 (5.51)		0.13 (1, 52)		.71		0.003		N/A		
	C (r)	64.97 (6.90)		65.08 (6.52)		67.83 (5.80)		67.92 (5.51)		2.11 (1, 52)		.15		0.04		N/A		
	ER (r)	.83 (1.24)		1.63 (2.21)		1.65 (1.32)		1.29 (1.20)		2.17 (1, 52)		.15		0.04		N/A		
**SA^k^**																		
	N (r)	39.47 (11.08)		42.92 (12.59)		47.30 (13.65)		45.27 (11.22)		3.11 (1, 52)		.08		0.06		N/A		
	C	38.97 (11.21)		41.35 (12.19)		46.47 (13.33)		43.46 (12.21)		8.26 (1, 52)		.006		0.14		EG > CG		
	ER (r)	1.43 (2.99)		3.53 (3.88)		1.72 (2.46)		4.63 (7.51)		1.85 (1, 52)		.18		0.03		N/A		
**CA^l^**																		
	N	7.90 (2.19)		7.46 (2.55)		9.07 (2.66)		8.65 (3.21)		0.09 (1, 52)		.76		0.002		N/A		
	C	6.73 (2.11)		5.42 (2.84)		8.07 (2.46)		6.35 (3.26)		1.45 (1, 52)		.23		0.03		N/A		
	ER (r)	14.57 (16.24)		30.32 (25.50)		10.70 (10.59)		27.08 (22.93)		8.40 (1, 52)		.005		0.14		EG < CG		
**SM^m^**																		
	N	28.60 (9.47)		33.04 (11.00)		31.63 (9.30)		35.27 (10.41)		0.04 (1, 52)		.85		0.001		N/A		
	C	27.70 (9.81)		31.31 (10.81)		30.47 (9.10)		33.19 (11.11)		0.01 (1, 52)		.92		0.0002		N/A		
	ER (r)	3.76 (5.98)		5.51 (6.07)		3.69 (4.75)		6.46 (11.15)		0.01 (1, 52)		.93		0.0001		N/A		
**CM^n^**																		
	N	13.43 (2.61)		13.42 (3.05)		14.27 (3.05)		13.92 (2.53)		0.01 (1, 52)		.91		0.0002		N/A		
	C	12.30 (2.58)		11.85 (2.92)		12.80 (2.71)		12.31 (2.82)		0.21 (1, 52)		.64		0.004		N/A		
	ER	8.54 (8.71)		11.25 (10.54)		9.65 (9.64)		11.86(9.64)		0.04 (1, 52)		.84		0.001		N/A		

^a^VMRT: Vandenberg and Kuse mental rotations test.

^b^GMC: general motor coordination (all measurements in seconds).

^c^N/A: not applicable.

^d^LC: locomotion circuit (Harre circuit test).

^e^DC: comparison of dot ensembles.

^f^N: total number of items performed.

^g^r: ANCOVA computed on ranks.

^h^C: number of correct responses.

^i^ER: error rate.

^j^NC: number comparison.

^k^SA: simple addition.

^l^CA: complex addition.

^m^SM: simple multiplication.

^n^CM: complex multiplication.

For all ANCOVA results, the estimated marginal means and corresponding 95% CIs are given in Multimedia Appendix 5.

## Discussion

### Principal Findings

This pilot interventional study aimed to verify whether DEx, involving mental rotation to perform different types of locomotion and interlimb coordination, may influence (1) mental rotation efficiency, (2) general motor coordination, and (3) math achievement (quantity comparisons, simple and complex additions, and multiplications) in an EG of adolescent students (Multimedia Appendix 6). This was done using a pretest-posttest design, including a CG involved in TEx and monitoring physical activity intensity levels and situational interest.

Noninstrumented observation suggested that the sessions proceeded as planned. This was corroborated by physical activity monitoring (Table 1), which also showed that MVPA timed values approached one-third of the daily 3600 seconds recommended for beneficial effects on adolescent health [54], that is, from 1137 (31.6%) seconds to 1372 (38.1%) seconds. The monitored amounts of moderate and vigorous physical activity were consistent with the expectations based on the planned sequences, which suggested that situational interest did not impede the physical activity required by DEx and TEx [59]. Only the scores for the triggered situational interest factor in session 5 significantly differed between groups, with a higher score in EG than in CG (large effect size), possibly due to the choice of the DEx and TEx activities and how they were perceived by the participants [58] (Table 2). ANCOVAs were performed controlling for this variable, considering the possible influence of situational interest on energy expenditure [59], thus exergaming task repetition and related learning [22].

The main ANCOVA results showed an advantage for EG after DEx over CG after TEx on mental rotation efficiency, the number of correct simple additions performed, and the error rate in complex addition (medium to large effect sizes). No other statistically significant ANCOVA results were found.

### Interpretation

#### Exergaming Influence on Mental Rotation Test Scores

Previous meta-analysis found that spatial ability (including mental rotation) may be improved by training [44], whereas in our study, DEx in EG, but not TEx in CG, was likely to elicit mental rotation training. The advantage for EG over CG regarding mental rotation found, once DEx and TEx were completed, may thus be due to mental rotation training in EG during DEx [44]. This influence of DEx on mental rotation in adolescents is consistent with the results of previous studies showing the influence on mental rotation of exergaming based on leg movements, but in adult patients with chronic stroke [74], and of certain physical activities (eg, wrestling vs endurance running) in healthy students, but which were practiced outside the exergaming framework [45].

Furthermore, in this study, DEx influence on mental rotation was found using a test [63] with tasks and stimuli (geometrical structures to be compared to a reference) different from mental rotation training tasks and stimuli (images of avatars and humans to be related to one’s own body). This supports that mental rotation training may elicit transfer to tasks and stimuli different from those used for training. Such a result, in line with those of previous studies obtained without physical activity during mental rotation training [75], contributes to fuel a long-standing and still running debate in the literature [76].

#### Exergaming Influence on General Motor Coordination

This study showed no significant difference regarding general motor coordination between EG and CG, once DEx and TEx were completed (Table 3). No difference was expected for sprinting, as neither DEx nor TEx trained sprinting capacities. Sprint performance times recorded in EG and CG during pretests and posttests (Table 3) showed that EG and CG had similar and stable potential to achieve the run parts of the locomotion circuit used to assess general motor coordination [52]. Thus, DEx and TEx showed similar influence on the performance of the other parts of the circuit, and the averaged performance improved by 3% (0.69/22.41 seconds) in EG and by 5% (1.22/22.42 seconds) in CG.

It remains that these variations did not lead to significant between-group differences in terms of ANCOVA results, while movement requirements differed between DEx and TEx. Considering that general motor coordination is a multidimensional construct [77], both DEx and TEx might have developed abilities influential on the performance of the locomotion circuit used for assessment. For example, DEx influence on mental rotation might have impacted this performance [52], but TEx may have developed other abilities with such an impact, particularly those related to the ability to react to a signal involved in moving to catch a ball [78]. On such a basis and considering the existing literature [23], further research remains needed to clarify whether, and under which conditions, exergaming might influence general motor coordination.

#### Exergaming Influence on Math Achievement

A previous study reported the positive influence of exergaming based on aerobic dance on the *global* score of a test assessing math achievement [36]. Our results confirmed such an influence of DEx when compared with TEx. However, this influence was found to be limited to peculiar mathematical tasks (Table 3). No significant differences between EG and CG were found regarding the “number tasks” [79] of quantity comparison (ie, with numbers and quantities but without requirements in formal calculation). On the other hand, mixed results were obtained regarding the “calculation tasks” [79] (ie, requiring the application of operation rules to numbers), with (1) no significant between-group differences in multiplications, and (2) a significant advantage for EG over CG regarding the number of simple additions correctly performed and the error rate in complex additions. Calculation of simple additions was thus faster in EG than in CG, without detrimental effect on the error rate, and the error rate in complex additions was lower in EG than in CG, without detrimental effect on calculation speed.

According to the literature, this advantage for EG might be limited to tasks primarily requiring procedural strategies for calculating, rather than strategies for retrieving memorized results [80]. In support of this possibility, additions, even simple (eg, 7+5), have been found to rely on procedural strategies more than simple multiplications (eg, 7 × 5), mainly based on retrieval strategies [80]. This explanation appears plausible, though we found no differences between EG and CG in terms of complex multiplications. Such multiplications may involve a mixture of simple multiplication and addition (eg, for adding the retrieved results of 2 × 7 and of 10 × 7, when calculating 12 × 7), that is, retrieval strategies, combined with calculation procedures [81,82]. Although EG had an advantage over CG in additions (Table 3), this might have elicited a weak impact on efficacy in complex multiplications. Overall, this is also consistent with the advantage in mental rotation efficiency for EG over CG found in our study (Table 3). Indeed, studies showed that mental rotation may improve calculation efficiency by promoting appropriate access to a representation of numbers as a mental number line [83], particularly involved during addition, but not multiplication [84].

### Limitations

Although our study helps to fill a gap in the literature concerning the possible influence of exergaming on academic performance, it is not without its limitations. One limitation is linked to the implementation of scientific research in a school environment. Notably, this did not allow us to allocate students randomly between EG and CG. Possible generalization of the results should also be questioned, especially as these results might depend on features of the exergaming sequences implemented, of the school of implementation, and of the students involved in the sequences. Confirmation of our results should also be sought using larger samples. Especially, in this pilot study, no formal correction for multiple comparisons was applied to the ANCOVA tests since applying strict corrections could have increased the risk of type II errors, given the sample size, the number of factors, and related outcomes [85]. Larger samples would also be needed to clarify the extent to which the selected DEx and TEx activities may modulate our results, by controlling for physical activity (eg, varying the levels of physical activity intensity in 2 or more DEx sequences, as well as in 2 or more TEx sequences), and situational interest [59]. Furthermore, larger samples would allow for proper examination of possible gender differences in the influence of exergaming on spatial abilities and math performance, ideally taking age into account [86,87]. Longer sequences than those used in this study could also be implemented for confirmation purposes [22].

### Perspectives

The results of this study encourage us to clarify whether mental rotation training through exergaming may influence math achievement. These results also encourage further investigation to determine if the influence of exergaming on the performance of arithmetical tasks is limited to tasks requiring procedural strategies for calculation. This also raises the question of whether such influence might depend on age, gender, or math experience. Analysis of task repetition and participants’ experience when exergaming might be useful in clarifying these points. Moreover, this study found no evidence of an effect of exergaming on general motor coordination, but a slight improvement in general motor coordination scores was observed in both exergaming sequences considered. This encourages further research to clarify the possible influence of exergaming on general motor coordination and its potential links to mental rotation training during exergaming. It would also be of interest to assess the potential added value of specific exergaming features (eg, avatar demonstration or options for managing exercise variety and progression) in achieving the effects observed in our study [88]. Teacher interventions directly linked to such features could complementarily be considered [23]. This assessment might be done by comparison with a conventional physical activity.

### Conclusion

The possible benefits of exergaming in schools have been little studied to date, except in relation to promoting physical activity and health [20]. Especially, no studies have yet been conducted to determine whether a given exergaming sequence may positively influence mental rotation, general motor coordination, and math achievement. For the first time, to our knowledge, our study provides support for the possibility that the same DEx sequence without math content may have a positive influence, in the same adolescent students, on performance in a mental rotation test and in tasks of addition. The results of this study encourage complementary investigations, especially to delimit the possible influence of practicing an exergame on math achievement and determine to what extent such influence might depend on mental rotation training, and possibly motor coordination training, during this practice. However, these results suggest that it would be appropriate to integrate exergames into an active learning approach at school, to promote both PE and math outcomes.

## Data Availability

For follow-up purposes, the datasets analyzed during this study are available from the corresponding author upon reasonable request [89].

## Appendix

Multimedia Appendix 1 Dance-based exergaming sequence (DEx).

Multimedia Appendix 2 Exergaming sequence based on precision ball-throwing (TEx).

Multimedia Appendix 3 Situational interest monitoring.

Multimedia Appendix 4 Pretests and posttests.

Multimedia Appendix 5 Estimated marginal means and 95% CIs.

Multimedia Appendix 6 CONSORT-EHEALTH (V 1.6.1) checklist.

## Abbreviations

ANCOVA: analysis of covariance
CG: control group
DEx: dance-based exergaming
EG: experimental group
MANOVA: multivariate analysis of variance
MVPA: moderate to vigorous physical activity
PE: physical education
TEx: precision ball-throwing exergaming
