# Supplementary material for: The Effects of Dance-Based Exergaming on Mental Rotation, General Motor Coordination, and Math Achievement in Adolescent Students: Nonrandomized Controlled Pilot Study
Source: JMIR Serious Games. 2026 Mar 19;14:e82610. doi: 10.2196/82610 (PMC13047359; doi:10.2196/82610)
Supplement: Multimedia Appendix 1 [file games_v14i1e82610_app1.pdf]

Dance-based exergaming sequence (DEx)

Overview of DEx

During DEx the exergame Grööve (available on the Play Lü® platform) was practiced by the subjects of the experimental group (EG) with the aim to learn a dance described in Figure S1.1.

Figure S1.1. Dance learned during the exergame Grööve.

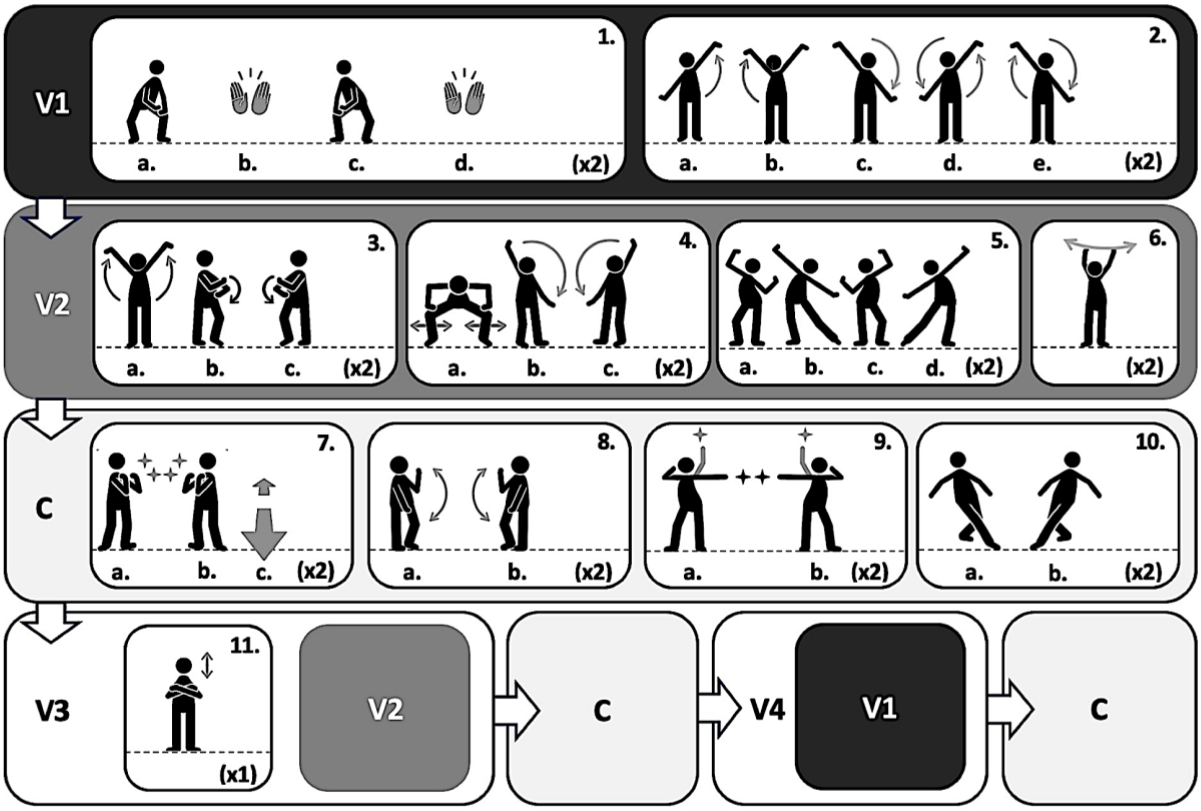

In this study, the exergame Grööve was used to teach the dance shown in Figure S1-1. during a sequence consisting of five weekly 45-minute sessions. The sequence was part of a study (pretest-posttest design) of dance-based exergaming influence (experimental group) on mental rotation, general motor coordination, and math achievement in adolescent students. This influence was compared to that of precision ball-throwing-based exergaming (control group).

The dance, shown by three avatars, was structured as function of a musical support (i.e., a song entitled "First thing") that comprises four verses (V1 to V4) and a chorus (C).

V1: Two series of body movements (1. and 2.), each series performed twice consecutively (x2).
- 1.: a. Flexion-extension of the legs (twice), hands on the left knee, b. Clapping twice, c. Idem a., but hands on the right knee, d. Idem b.
- 2.: a. Standing up, raising left arm in the frontal plane, b. Raising right arm as in a., c. Lowering left arm, d. Raising left arm while lowering right arm, e. Lowering left arm while raising right arm.

V2: Four series of movements (3. to 6.; x2).
- 3.: a. Raising arms simultaneously in the frontal plane, b. Arm rolls on left side, c. Idem b., but on right side.
- 4.: a. Hands on knees, flexing the legs, spreading, and tightening the knees, b. Standing up left arm down and right arm up, then reaching the left hand with the right one, c. Idem b., interchanging the arms.
- 5.: a. Flexing the trunk on the left while flexing the arms at 90° in the frontal plane, b. Outstretching the arms and sliding the outstretched left leg to the left, c. Idem a., but flexing the trunk on the right, d. Idem b., but sliding the right leg to the right.
- 6.: Arms over the head, shaking the arms from the left to the right and from the right to the left.

C: Four series of movements (7. to 10.; x2), performed after V2, V3, and V4.
- 7.: a. Flexing the arms towards the left side and snapping fingers, b. Idem a., but on right side, c. Walking forward, then backward.
- 8.: a. Left quarter turn, then flexing left arm and stretching right arm, and vice versa (twice), b. Right three-quarter turn, and idem a.
- 9.: a. In the frontal plane, maintaining right arm flexed while extending left arm horizontally, then vertically, b. Idem a. interchanging the arms.
- 10.: a. Standing up crossing the outstretched left leg in front of the right leg, b. idem a., interchanging the legs.

V3: One series of movements (11.) performed once (x1), then reiteration of the V2 movements.
- 11.: Arms crossed over chest, flexing, and extending the neck twice.

V4: Reiteration of the V1 movements.

The content of the sessions was determined based on the structure of the dance to be mastered (see: Figure S1.1), which allowed both variety and progressivity during learning:

- Session 1: Learn the body movements (BMs) for the first and second verses.
- Session 2: Repeat the BMs for the first and second verses, then learn the BMs for the chorus.
- Session 3: Repeat the BMs for the first and second verses and the chorus, then learn the BMs for the third verse (similar to the second) and the fourth verse (identical to the first).
- Session 4: Repeat the different parts of the dance, then, progressively, the entire dance under facilitating conditions (e.g., with slow speed of movement).
- Session 5: Idem session 4, then repeat the dance under final conditions.

Implementation of the sessions

Each session of DEx was beginning by a ~5-minute warm-up including bodily movements performed with music (e.g., handball dribbling to beat off the music, and maintaining a semi-squatted position when the music was stopped).

During the sessions 1 to 3, each part of the dance (for each verse and the chorus) has been decomposed into subparts (e.g., for the first verse, flexion-extension of the legs with both hands on the left knee and clapping hands; see: Figure S1.1). Each subpart has been first practiced separately from any other. Once mastered by the subjects, two consecutive subparts have been repeated without discontinuity, and so on. For each learning step, the dance movements were shown and reproduced under facilitating conditions; then, the facilitating elements were progressively removed. This has been done using specific options available to practice Grööve, that allowed repetitions: (1) at slow speed (x0.50) with a metronome and without music, (2) at medium speed (x0.75) with the same sound conditions, (3) at normal speed (x1.00) with the same sound conditions, (4) at normal speed with both metronome and music, then (4) at normal speed with music only. Such options have been also used to allow progressive repetition of the entire dance from session 4 to session 5.

At the end of each session, formative evaluation was done based on videos and freeze-frames of the subjects dancing to highlight the progress made and the prospects for the next session.
