# Supplementary material for: The Effects of Dance-Based Exergaming on Mental Rotation, General Motor Coordination, and Math Achievement in Adolescent Students: Nonrandomized Controlled Pilot Study
Source: JMIR Serious Games. 2026 Mar 19;14:e82610. doi: 10.2196/82610 (PMC13047359; doi:10.2196/82610)
Supplement: Multimedia Appendix 2 [file games_v14i1e82610_app2.pdf]

Exergaming sequence based on precision ball-throwing (TEx)

Overview of TEx

During TEx, the subjects of the control group (CG) were involved in exergaming based on precision ball-throwing using eight applications available on the Lü platform that are described in Figure S2.1.

Figure S2.1. Applications used during the sequence based on precision ball-throwing (TEx).

Application	Screen	Description
**Target**	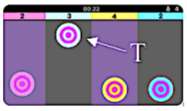	Up to six teams can compete simultaneously, players throw a ball to hit colored targets (T; one color per team). *For each team, targets are either placed in column or scattered; the size of the targets can be adjusted and presentation duration, lengthened or shortened.*
**Galactic**	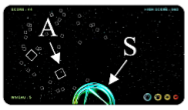	One team at a time; players defend a spaceship (S) by throwing balls to destroy asteroids (A). Points are gained when hitting a white asteroid and bonus points, when hitting a colored one. *Four difficulty levels (frequency and speed of asteroids).*
**Relé**	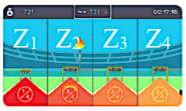	Up to four teams, partners passing a ball from the furthest to the closest to the screen who then throw it into the team zone ($Z_{1\text{-}4}$), catches it and passes it to the furthest from the screen. Players' positions change after a defined number of trials. *Cooperative or competitive mode; passing distance adjustable.*
**Spörts**	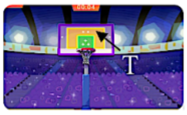	Practice of sports such as soccer, or basketball, based on shooting at a virtual basket in the present study. *Adjustable dimensions and height of the target. The task can also be made easier by adding colored areas to be aimed (T) that allow to gain points.*
**Germ**	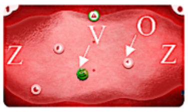	Two-team game, players throw a ball to direct a moving virus (V) towards the adverse zone (Z). Each impact of the ball on the screen creates temporary obstacle (O) on which V may bounce back. *Number of points to win the match to be selected (from three to nine).*
**Vïka**	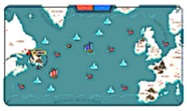	Two-team game; players throw a ball to answer questions (targets with possible answers), create wind to propel a boat to cities in the Vïka Kingdom and keep the villagers safe from a monster. *Up to five balls per team.*
**Lüvia**	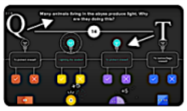	Two to four teams, players answer questions (Q) by selecting the target (T) that indicates their answer and throwing a ball to hit it. Possible break times practicing dance movements (option not used in the present study). *Contents and difficulty of the questions adaptable to the players.*
**Brüsh**	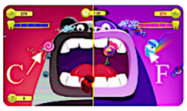	Up to two teams, players throw a ball to hit sweets (C) before they reach the teeth of an avatar, avoiding touching dental floss (F) present among the candies. *Playable in cooperative or competitive mode with four difficulty levels (speed of movement of candies and dental floss).*

This study (pretest-posttest design) aimed to determine the influence of a dance-based exergaming sequence (five weekly 45-minute sessions) on mental rotation, general motor coordination, and math achievement in adolescent students. This influence was compared to that of a sequence (TEx) precision ball-throwing-based exergaming (control group). Different applications were used during TEx that allowed variety in the practice of precision ball-throwing. The practice difficulty was also progressive thanks to the possibilities offered by each application (indicated in *italic* above). In each application it was also possible to shorten and lengthen the distance between the player throwing a ball and the target. To be noted: 1. Relé's practice has been specifically organized to promote ball pass and throwing, 2. In both Vïka and Lüvia, the questions that needed to be answered did not involve any mathematical content and did not require any mental rotation (questions determined from the available options).

Each session of TEx began with a ~5mn warm-up identical to that performed by the subjects of the experimental group involved in a dance-based exergaming sequence (see: Multimedia Appendix 1). This warm-up was followed by exergaming using three applications (~12

minutes each) including short instructions (rules, practice conditions), verification of instructions application, and task difficulty management.

During each session, the three applications used among eight possible (see: Figure S2.1) were:

- Session 1: Target, then Galactic, and finally Relé.
- Session 2: Target, then Galactic, and finally Spörts.
- Session 3: Germ, then Vïka, and finally Relé.
- Session 4: Lüvia, then Brüsh, and finally Spörts.
- Session 5: Lüvia, then Spörts, and finally Vïka.

Each session included applications focused on precision ball-throwing that were used more than once during TEx (Target, Galactic, Relé, Vïka, and Lüvia were used twice, and Spörts, three times). Two applications were used only once, that is, Germ and Brüsh that may be considered as variations of Galactic. This made it possible to manage the progression of difficulty, with increased difficulty in ball throwing, for example, increased speed of asteroid movement in Galactic (see: Figure S2.1), from one session to the next.

Implementation of sessions

The conditions of practice were varying depending two main features, each depending on a choice between two alternatives (Alt), with:

- Alt-1: the subjects were either (1) divided into teams to compete, or (2) brought together to cooperate in achieving a given goal.
- Alt-2: the subjects had either to (1) pass a ball one to another before the last receiver threw it at a virtual target, or (2) play a non-digital game based on precision ball throwing, with rules allowing them to leave this game temporarily and make ball throws at targets during an exergame played in parallel.

For example, during lesson 1, the subjects played Galactic (see: Figure S2.1) cooperatively to protect a spaceship throwing balls to destroy asteroids before they hit this spaceship. Subgroups were formed, the members of each subgroup were aligned in column in front of the screen, and two balls were given to each subgroup. The subjects had to pass each ball one to the other until the one at the head of the column threw it to destroy an asteroid and protect the spaceship. Consequently, these subjects were placed in conditions of practice including both [Alt-1.(2)] and [Alt-2.(1)].

At the end of each session, an oral report was done on the progress made and the outlook for the next session.
