# Supplementary material for: The Effects of Dance-Based Exergaming on Mental Rotation, General Motor Coordination, and Math Achievement in Adolescent Students: Nonrandomized Controlled Pilot Study
Source: JMIR Serious Games. 2026 Mar 19;14:e82610. doi: 10.2196/82610 (PMC13047359; doi:10.2196/82610)
Supplement: Multimedia Appendix 3 [file games_v14i1e82610_app3.pdf]

Situational Interest Monitoring

Measurement

Situational interest, as conceived in the field of educational psychology [1-3], was assessed in each subject of the experimental and control groups, involved in sequences (five 45-minute weekly sessions) of dance-based exergaming and exergaming based on precision ball-throwing respectively. Assessment was done directly after the sessions 3 and 5 of these sequences, using the French situational interest questionnaire [4]. This 12-item questionnaire (see: Table S3.1) contains three factors: (1) triggered situational interest (participant's perception of the game demanding full attention), (2) maintained situational interest feeling (to what extent the activities were found enjoyable), and (3) maintained situational interest value (importance allocated to success during the game).

Table S3.1. Situational interest questionnaire.

Factors	Questions
Triggering situational interest	1. What we were learning during exergames had a complexity level adapted to my abilities
	2. What we were learning during exergames required concentration
	3. What we were learning during exergames had a difficulty level adapted to my abilities
	4. What we were learning during exergames demanded my high attention
Maintained situational interest feeling	5. What we did during exergames was enjoyable for me
	6. I wanted to know more about what we did during exergames
	7. It was fun for me to practice the exergames today
	8. I wanted to further explore what we did during exergames
Maintained situational interest value	9. It was important for me to succeed in exergames during this lesson
	10. What we were learning during exergames was interesting for me
	11. I find it important to do well in exergames during this lesson
	12. What we were learning during exergames seemed useful for what I do outside of school

The French version of this questionnaire was administered immediately after the sessions 3 and 5 of two exergaming sequences, which consisted of five weekly 45-minute sessions. This was part of a study of the effects of dance-based exergaming (experimental group), compared to exergaming based on precision ball throwing (control group), on mental rotation, general motor coordination, and math achievement in adolescent students.

To complete each questionnaire (Table S3.1), the subjects had to rate statements on a five-point Likert scale [from 1 (strongly disagree) to 5 (strongly agree)]. This led to obtain scores for each factor, these scores ranging from 1 (lowest possible score) to 5 (highest possible score).

References

1. Renninger KA, Hidi SE. The power of interest for motivation and engagement. New York: Routledge; 2017. ISBN: 9781138779792
2. Schiefele U. Situational and individual interest. In: Wentzel KR, Wigfield A. Handbook of motivation at school. 1st ed. New York: Routledge; 2009, p. 197-222. ISBN: 0805862846
3. Hidi SE, Renninger KA. The four-phase model of interest development. Educ Psychol. 2006;41(2):111-127. [doi: 10.1207/s15326985ep4102_4]
4. Roure C. Clarification du construit de l'intérêt en situation en éducation physique. STAPS. 2020;130(4):61-77. [doi: 10.3917/sta.130.0061]
