# Supplementary material for: The Effects of Dance-Based Exergaming on Mental Rotation, General Motor Coordination, and Math Achievement in Adolescent Students: Nonrandomized Controlled Pilot Study
Source: JMIR Serious Games. 2026 Mar 19;14:e82610. doi: 10.2196/82610 (PMC13047359; doi:10.2196/82610)
Supplement: Multimedia Appendix 4 [file games_v14i1e82610_app4.pdf]

Pretests and Posttests

Mental Rotation

The subjects completed the French version [1] of Vandenberg and Kuse's mental rotations test [2]. This paper-and-pencil test was explained to the subjects and three trials were performed to ensure a correct understanding of the test (Figure S4.1).

Figure S4.1. Vandenberg and Kuse mental rotations test.

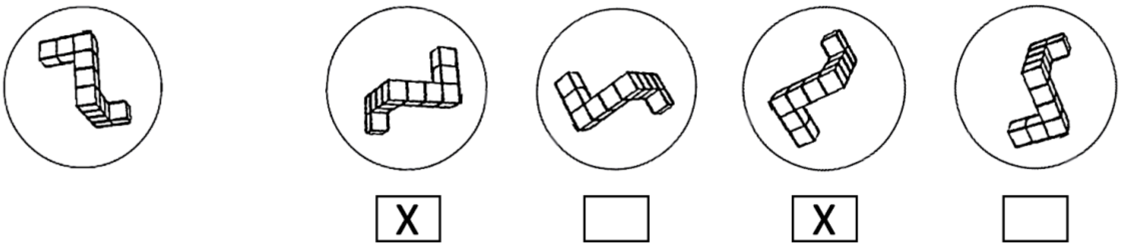

Example of items inspired by Vandenberg and Kuse's mental rotations test (produced using Blender 2.76). This test was administered (pretest and posttest) as part of a study of the influence of dance-based exergaming (experimental group), compared with precision ball-throwing-based exergaming (control group), on mental rotation, general motor coordination, and math achievement in adolescent students. Regarding mental rotation, the used version of the test included 20 items of this type, each showing five drawings of asymmetric structures made of 10 cubes. The left drawing was a reference. Two of the four other drawings showed the same structure as the reference after a rotation in space. The subjects were asked to find these two drawings. The scoring procedure, aiming to discourage random responses, was explained (two points for two correct responses, one point when only one response was given and was a correct one, and zero point otherwise).

General Motor Coordination

The subjects performed a locomotion circuit called Harre circuit test in the literature (e.g., [3]), aimed at assessing general motor coordination [4]. After a warm-up, the circuit tasks (Figure S4.2) were described by the experimenter. Thereafter, each subject performed one trial on the circuit; each error was indicated. Before the test, the subjects were asked to perform the circuit as fast as possible, but without error. Only the chronometric performance of a correctly executed circuit was registered. In the event of an error, a second attempt was done. This allowed each subject to complete the circuit correctly.

Figure S4.2. Locomotion circuit used to assess general motor coordination.

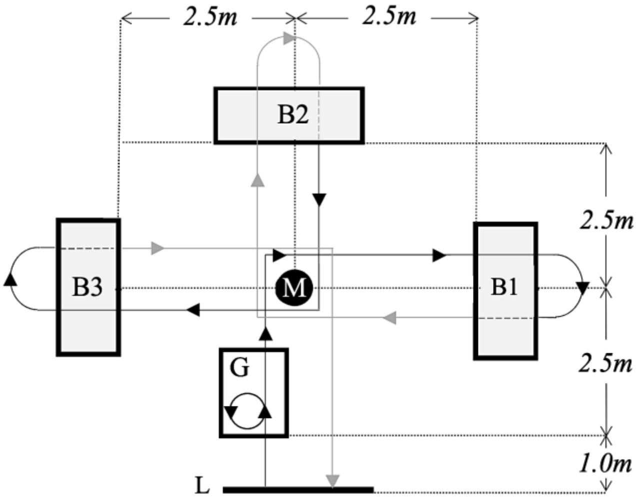

The Harre circuit test test was administered (pretest and posttest) as part of a study of the influence of dance-based exergaming (experimental group), compared with precision ball-throwing-based exergaming (control group), on mental rotation, general motor coordination, and math achievement in adolescent students. The installation of the Harre Circuit Test requires: a line (L) drawn on the ground, a gymnastic mat (G), a medicine-ball (M) and three benches (B1-3) under which it is possible to crawl. The subjects were required: (1) from L to G (L-G), stand behind L and react to an acoustic signal to perform a forward roll on G, (2) G-B1, run to M, go around M to the right at ~90°, run to B1, jump over B1, and crawl under it, (3) B1-B2, idem, with B2 instead of B1, (4) B2-B3, idem, with B3, and (5) B3-L, run to M, go around M at ~90°, and run to L.

On the other hand, it was considered that any test to assess general motor coordination involves determinants of physical fitness [5]. Especially, the performance on the Harre circuit test has been found to be influenced by sprinting ability [6]. Consequently, a sprint race (20m), initiated by an acoustic signal, was performed separately from the locomotion circuit, after a warm-up. All performances were measured using the same wireless timing system (Race link Racing System; Brower Timing Systems, Draper, UT, USA).

Math Achievement

Quantity Comparisons

The subjects performed two separate series of quantity comparisons. For this purpose, 72 comparisons of two ensembles of dots (Figure S4.3) and 72 comparisons of two numbers (Figure S4.4) were designed based on a previous study [7].

Figure S4.3. Comparisons of ensembles of dots.

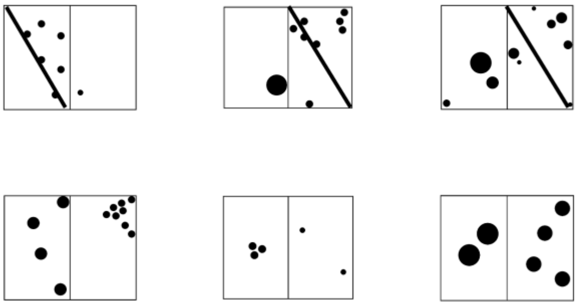

A test of quantity comparison, based on ensembles of dots, was administered (pretest and posttest) as part of a study of the influence of dance-based exergaming (experimental group), compared with precision ball-throwing-based exergaming (control group), on mental rotation, general motor coordination, and math achievement in adolescent students. Figure S4.3. shows examples of test items involving comparing ensembles of dots. The subjects were asked to find the larger of two quantities.

Figure S4.4. Number comparisons.

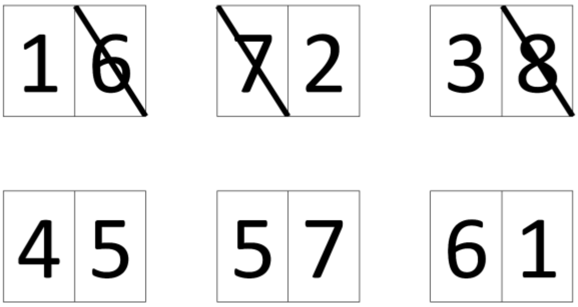

A test of quantity comparison, based on ensembles of dots, was administered (pretest and posttest) as part of a study of the influence of dance-based exergaming (experimental group), compared with precision ball-throwing-based exergaming (control group), on mental rotation, general motor coordination, and math achievement in adolescent students. Figure S4.4. shows examples of test items involving number comparison. The subjects were asked to find the larger of two quantities.

For both series of comparisons (Figures S4.3 and S4.4), each quantity was between one and nine. Three trials were done to check task comprehension. Subjects were asked to make as many accurate comparisons as possible during 60 sec.

Mental Calculation

The subjects performed four separate series of mental calculations. For this purpose, 139 additions of two single-digit numbers (e.g., 6+2=), 60 additions of three double-digit numbers (e.g., 21+52+67=), 64 multiplications of two single-digit numbers (e.g., 2x8=), and 60 multiplications of a double-digit number with a single-digit number (e.g., 25x9=) were designed in accordance with a previous study [8]. Additions and multiplications of two single-digit numbers were considered simple and the other calculations, complex [9,10]. For each

calculation, three trials were done to ensure correct comprehension of the task; the subjects were asked to perform as many accurate calculations as possible during 105 sec.

In each quantity comparison and mental calculation task, we considered, for analysis purposes, the total number of processed items, the number of correct results, and the error rate.

References

1. Albaret JM, Aubert E. Étalonnage 15-19 ans du test de rotation mentale de Vandenberg. Evol. Psychom. Jan 1, 1996;8(34):205-215.
2. Vandenberg SG, Kuse AR. Mental rotation, a group test of three-dimensional spatial visualization. Percept Mot Skills. Dec, 1978;47(2):599-604. [doi: 10.2466/pms.1978.47.2.599] [Medline: 724398]
3. Dallolio L, Ceciliani A, Sanna T, Garulli A, Leoni E. Proposal for an enhanced physical education program in the primary school: evaluation of feasibility and effectiveness in improving physical skills and fitness. J Phys Act Health. Oct, 2016;13(10):1025-1034. [doi: 10.1123/jpah.2015-0694] [Medline: 27172612]
4. Harre D., Barsch J. Principles of sports training: Introduction to the theory and methods of training. 1st edition. Berlin: Sportverlag; 1982. ISBN: 3210031
5. Vandorpe B, Vandendriessche J, Lefevre J, et al. The körperkoordinations test für kinder: reference values and suitability for 6-12-year-old children in Flanders. Scand J Med Sci Sports. Jun, 2011;21(3):378-388. [doi: 10.1111/j.1600-0838.2009.01067.x] [Medline: 20136753]
6. Hoyek N, Champely S, Collet C, Fargier P, Guillot A. Is mental rotation ability a predictor of success for motor performance? J Cogn Dev. 2014;15(3):495-505. [doi: 10.1080/15248372.2012.760158]
7. Nosworthy N, Bugden S, Archibald L, Evans B, Ansari D. A two-minute paper-and-pencil test of symbolic and nonsymbolic numerical magnitude processing explains variability in primary school children's arithmetic competence. PLoS One. Jul 2, 2013;8(7):e67918. [doi: 10.1371/journal.pone.0067918] [Medline: 23844126]
8. Vogel SE, Haigh T, Sommerauer G, et al. Processing the order of symbolic numbers: a reliable and unique predictor of arithmetic fluency. J Numer Cogn. Dec 22, 2017;3(2):288-308. [doi: 10.5964/jnc.v3i2.55]
9. Thevenot C, Castel C, Fanget M, Fayol M. Mental subtraction in high- and lower skilled arithmetic problem solvers: verbal report versus operand-recognition paradigms. J Exp Psychol Learn Mem Cogn. Sep, 2010;36(5):1242-1255. [doi: 10.1037/a0020447] [Medline: 20804294]
10. Tschentscher N, Hauk O. How are things adding up? Neural differences between arithmetic operations are due to general problem solving strategies. Neuroimage. May 15, 2014;92:369-380. [doi: 10.1016/j.neuroimage.2014.01.061] [Medline: 24525170]
