# Supplementary material for: The Effects of Dance-Based Exergaming on Mental Rotation, General Motor Coordination, and Math Achievement in Adolescent Students: Nonrandomized Controlled Pilot Study
Source: JMIR Serious Games. 2026 Mar 19;14:e82610. doi: 10.2196/82610 (PMC13047359; doi:10.2196/82610)
Supplement: Multimedia Appendix 5 [file games_v14i1e82610_app5.pdf]

Estimated marginal means and 95% CIs

Physical activity intensity levels

Table 1 – Estimated marginal means and 95% CIs of physical activity intensity levels

Dependent variable	Group	Mean (SE)	95% CI
S3 sec[a]			
Moderate	EG[b] (n=30)	739.67 (25.24)	689.05-790.29
	CG[c] (n=26)	1060.77 (27.12)	1006.40-1115.14
Vigorous	EG (n=30)	336.67 (20.52)	295.53-377.80
	CG (n=26)	283.65 (22.04)	239.46-327.84
MVPA[d]	EG (n=30)	1180.67 (42.90)	1094.66-1266.67
	CG (n=26)	1419.23 (46.08)	1326.85-1511.61
S3 %[e]			
Moderate	EG (n=30)	35.9 (1.0)	33.9-37.9
	CG (n=26)	42.0 (1.1)	39.9-44.1
Vigorous	EG (n=30)	16.4 (0.9)	14.5-18.2
	CG (n=26)	12.4 (1.0)	10.4-14.4
MVPA	EG (n=30)	57.4 (1.7)	54.1-60.7
	CG (n=26)	56.1 (1.8)	52.5-59.6
S5 sec[f]			
Moderate	EG (n=30)	724.67 (24.59)	675.37-773.97
	CG (n=26)	1035.00 (26.41)	982.04-1087.96
Vigorous	EG (n=30)	322.67 (16.64)	289.30-356.04
	CG (n=26)	220.96 (17.88)	185.12-256.81
MVPA	EG (n=30)	1189.33 (41.70)	1105.73-1272.93
	CG (n=26)	1311.92 (44.79)	1222.12-1401.72
S5 %[g]			
Moderate	EG (n=30)	32.2 (0.9)	30.3-34.0
	CG (n=26)	40.9 (0.1)	38.9-42.9
Vigorous	EG (n=30)	14.3 (0.8)	12.8-15.9
	CG (n=26)	9.9 (0.8)	8.2-11.6
MVPA	EG (n=30)	52.7 (1.6)	49.5-55.9
	CG (n=26)	51.8 (1.7)	48.3-55.2

Physical activity intensity levels were monitored during sessions 3 and 5 of two exergaming sequences (five weekly sessions of 45 minutes each). This monitoring was part of a study (pretest-posttest design) of dance-based exergaming influence (experimental group), compared to precision ball-throwing-based exergaming (control group), on mental rotation, general motor coordination, and math achievement in adolescent students. Physical activity monitoring was carried out to verify that the exergaming sessions had been implemented as planned. In Table 1, the statistically significant between-group differences regarding physical intensity levels (MANOVAs) are indicated in green. The estimated marginal means of the intensity levels of physical activity measured in each group during sessions 3 and 5 are given, as well as the 95% CIs.

[a]S3 sec: intensity levels of physical activity in seconds during session 3

[b]EG: experimental group

[c]CG: control group

[d]MVPA: moderate-to-vigorous physical activity

[e]S3 %: intensity levels of physical activity in percentage of the total physical activity time during session 3

[f]S5 sec: intensity levels of physical activity in seconds during session 5

[g]S5 %: intensity levels of physical activity in percentage of the total physical activity time during session 5

Situational interest factors

Table 2 - Estimated marginal means and 95% CIs of situational interest factors

Dependent variable	Group	Mean (SE)	95% CI
SI-S3[a]			
TSI[b]	EG[c] (n=30)	3.52 (0.24)	3.04-4.00
	CG[d] (n=26)	2.89 (0.26)	2.38-3.41
MSIF[e]	EG (n=30)	2.63 (0.20)	2.22-3.04
	CG (n=26)	2.21 (0.22)	1.77-2.65
MSIV[f]	EG (n=30)	2.59 (0.20)	2.18-3.00
	CG (n=26)	2.16 (0.22)	1.72-2.60
SI-S5[g]			
TSI	EG (n=30)	3.49 (0.23)	3.03-3.95
	CG (n=26)	2.47 (0.25)	1.98-2.97
MSIF	EG (n=30)	2.34 (0.20)	1.94-2.74
	CG (n=26)	2.02 (0.21)	1.59-2.44
MSIV	EG (n=30)	2.42 (0.20)	2.01-2.83
	CG (n=26)	2.08 (0.22)	1.64-2.52

Situational interest was assessed directly after the sessions 3 and 5 of two exergaming sequences (five weekly sessions of 45 minutes each). This monitoring was part of a study (pretest-posttest design) of dance-based exergaming influence (experimental group), compared to precision ball-throwing-based exergaming (control group), on mental rotation, general motor coordination, and math achievement in adolescent students. Situational interest was assessed to control for possible between-group differences likely to have influenced subjects' activity. In Table 1, the statistically significant between-group differences regarding the situational interest factors (MANOVAs) are indicated in green. The estimated marginal means of each factor of situational interest measured during sessions 3 and 5 are given, as well as the 95% CIs.

[a]SI-S3: situational interest in session 3
[b]TSI: triggered SI
[c]EG: experimental group
[d]CG: control group
[e]MSIF: maintained SI feeling
[f]MSIV: maintained SI value
[g]SI-S5: SI in session 5

Mental rotation, general motor coordination, and math skills

Table 3 - Estimated marginal means and 95% CIs of scores on tests of mental rotation, general motor coordination, quantity comparisons, and calculations

Dependent variable		Group	Mean (SE)	95% CI
**VMRT**[a]				
	Score	EG[b] (n=30)	20.50 (1.19)	18.11-22.88
		CG[c] (n=26)	15.89 (1.29)	13.31-18.47
**GMC**[d]				
	Sprint	EG (n=30)	3.65 (0.44)	3.55-3.73
		CG (n=26)	3.63 (0.41)	3.54-3.71
	LC[e]	EG (n=30)	21.76 (0.43)	20.90-22.63
		CG (n=26)	21.16 (0.46)	20.22-22.08
**DC**[f]				
	N[g] (r)[h]	EG (n=30)	27.11 (2.38)	22.33-31.88
		CG (n=26)	30.11 (2.58)	24.94-35.28
	C[i] (r)	EG (n=30)	28.35 (2.51)	23.32-33.38
		CG (n=26)	28.67 (2.72)	23.22-34.12
	ER[j]	EG (n=30)	8.86 (1.06)	6.74-10.98
		CG (n=26)	8.39 (1.14)	6.10-10.69
**NC**[k]				
	N (r)	EG (n=30)	29.02 (1.99)	25.03-33.00
		CG (n=26)	27.90 (2.15)	23.59-32.21
	C (r)	EG (n=30)	31.15 (2.56)	26.02-36.28
		CG (n=26)	25.44 (2.76)	19.89-30.99
	ER (r)	EG (n=30)	31.65 (2.98)	25.67-37.63
		CG (n=26)	24.87 (3.22)	18.39-31.34
**SA**[l]				
	N (r)	EG (n=30)	31.65 (2.98)	25.67-37.63
		CG (n=26)	24.87 (3.22)	18.39-31.34
	C	EG (n=30)	47.40 (1.13)	45.12-49.67
		CG (n=26)	42.40 (1.23)	39.93-44.84
	ER (r)	EG (n=30)	25.46 (3.04)	19.37-31.56
		CG (n=26)	32.00 (3.30)	25.38-38.62
**CA**[m]				
	N	EG (n=30)	8.81 (0.29)	8.23-9.39
		CG (n=26)	8.95 (0.31)	8.32-9.58
	C	EG (n=30)	7.57 (0.35)	6.86-8.28
		CG (n=26)	6.92 (0.38)	6.14-7.68
	ER (r)	EG (n=30)	22.60 (2.81)	16.96-28.23
		CG (n=26)	35.31 (3.04)	29.20-41.41
**SM**[n]				
	N	EG (n=30)	33.45 (1.05)	31.37-35.57
		CG (n=26)	33.15 (1.13)	30.88-35.43
	C	EG (n=30)	31.81 (1.10)	29.60-34.01
		CG (n=26)	31.64 (1.19)	29.26-34.03
	ER (r)	EG (n=30)	28.32 (3.00)	22.29-34.34
		CG (n=26)	28.71 (3.25)	22.18-35.24
**CM**[o]				
	N	EG (n=30)	14.13 (0.35)	13.43-14.83
		CG (n=26)	14.07 (0.38)	13.32-14.83
	C	EG (n=30)	12.45 (0.36)	11.73-13.17
		CG (n=26)	12.71 (0.39)	11.93-13.49
	ER	EG (n=30)	10.91 (1.60)	7.69-14.12
		CG (n=26)	10.40 (1.73)	6.92-13.88

Study (pretest-posttest design) of the influence of dance-based exergaming (experimental group), compared to exergaming based on precision ball throwing (control group), on mental rotation, general motor coordination (sprint and locomotor circuit), and math achievement (quantity comparisons and mental calculations) in adolescent students. In Table 3, statistically significant differences (ANCOVAs) are indicated in green. The estimated marginal means of posttest scores and 95% CIs are given.

[a]VMRT: Vandenberg and Kuse mental rotations test
[b]EG: experimental group
[c]CG: control group
[d]GMC: general motor coordination (all measurements in sec)
[e]LC: locomotion circuit (Harre circuit test)
[f]DC: comparison of dot ensembles
[g]N: total number of items performed
[h](r): ANCOVA computed on ranks
[i]C: number of correct responses
[j]ER: error rate
[k]NC: number comparison
[l]SA: simple addition
[m]CA: complex addition
[n]SM: simple multiplication
[o]CM: complex multiplication
