# Supplementary material for: The Effects of Dance-Based Exergaming on Mental Rotation, General Motor Coordination, and Math Achievement in Adolescent Students: Nonrandomized Controlled Pilot Study
Source: JMIR Serious Games. 2026 Mar 19;14:e82610. doi: 10.2196/82610 (PMC13047359; doi:10.2196/82610)
Supplement: Multimedia Appendix 6 [file games_v14i1e82610_app6.pdf]

# CONSORT-EHEALTH (V 1.6.1) - Submission/Publication Form

The CONSORT-EHEALTH checklist is intended for authors of randomized trials evaluating web-based and Internet-based applications/interventions, including mobile interventions, electronic games (incl multiplayer games), social media, certain telehealth applications, and other interactive and/or networked electronic applications. Some of the items (e.g. all subitems under item 5 - description of the intervention) may also be applicable for other study designs.

The goal of the CONSORT EHEALTH checklist and guideline is to be
a) a guide for reporting for authors of RCTs,
b) to form a basis for appraisal of an ehealth trial (in terms of validity)

CONSORT-EHEALTH items/subitems are MANDATORY reporting items for studies published in the Journal of Medical Internet Research and other journals / scientific societies endorsing the checklist.

Items numbered 1., 2., 3., 4a., 4b etc are original CONSORT or CONSORT-NPT (non-pharmacologic treatment) items.
Items with Roman numerals (i., ii, iii, iv etc.) are CONSORT-EHEALTH extensions/clarifications.

As the CONSORT-EHEALTH checklist is still considered in a formative stage, we would ask that you also RATE ON A SCALE OF 1-5 how important/useful you feel each item is FOR THE PURPOSE OF THE CHECKLIST and reporting guideline (optional).

Mandatory reporting items are marked with a red *.
In the textboxes, either copy & paste the relevant sections from your manuscript into this form - please include any quotes from your manuscript in QUOTATION MARKS,
or answer directly by providing additional information not in the manuscript, or elaborating on why the item was not relevant for this study.

YOUR ANSWERS WILL BE PUBLISHED AS A SUPPLEMENTARY FILE TO YOUR PUBLICATION IN JMIR AND ARE CONSIDERED PART OF YOUR PUBLICATION (IF ACCEPTED).
Please fill in these questions diligently. Information will not be copyedited, so please use proper spelling and grammar, use correct capitalization, and avoid abbreviations.

DO NOT FORGET TO SAVE AS PDF _AND_ CLICK THE SUBMIT BUTTON SO YOUR ANSWERS ARE IN OUR DATABASE !!!

Citation Suggestion (if you append the pdf as Appendix we suggest to cite this paper in the caption):
Eysenbach G, CONSORT-EHEALTH Group
CONSORT-EHEALTH: Improving and Standardizing Evaluation Reports of Web-based and Mobile Health Interventions
J Med Internet Res 2011;13(4):e126

URL: http://www.jmir.org/2011/4/e126/
doi: 10.2196/jmir.1923
PMID: 22209829

**fargier.patrick@gmail.com** Changer de compte

Non partagé

Brouillon enregistré

* Indique une question obligatoire

Your name *

First Last

Patrick Fargier

Primary Affiliation (short), City, Country *

University of Toronto, Toronto, Canada

University of Teacher Education, Lausanne, Sw

Your e-mail address *

abc@gmail.com

patrick.fargier@hepl.ch

Title of your manuscript *

Provide the (draft) title of your manuscript.

Effect of Dance-Based Exergaming on Mental Rotation, General Motor Coordination, and Math Achievement in Adolescent Students: Pilot Interventional Study

Name of your App/Software/Intervention *

If there is a short and a long/alternate name, write the short name first and add the long name in brackets.

Play Lü (Lü Interactive Playground)

Evaluated Version (if any)

e.g. "V1", "Release 2017-03-01", "Version 2.0.27913"

Votre réponse

Language(s) *

What language is the intervention/app in? If multiple languages are available, separate by comma (e.g. "English, French")

English, French

URL of your Intervention Website or App

e.g. a direct link to the mobile app on app in appstore (itunes, Google Play), or URL of the website. If the intervention is a DVD or hardware, you can also link to an Amazon page.

Votre réponse

URL of an image/screenshot (optional)

Votre réponse

Accessibility *

Can an enduser access the intervention presently?

- ○ access is free and open
- ○ access only for special usergroups, not open
- ○ access is open to everyone, but requires payment/subscription/in-app purchases
- ○ app/intervention no longer accessible
- ◉ Autre : Available only with the Play Lü platform for playing exergames

Primary Medical Indication/Disease/Condition *

e.g. "Stress", "Diabetes", or define the target group in brackets after the condition, e.g. "Autism (Parents of children with)", "Alzheimers (Informal Caregivers of)"

Active learning and physical activity promotior

Primary Outcomes measured in trial *

comma-separated list of primary outcomes reported in the trial

Mental rotation, General motor coordination, M

Secondary/other outcomes

Are there any other outcomes the intervention is expected to affect?

Moderate-to-vigorous physical activity, Situational interest

Recommended "Dose" *

What do the instructions for users say on how often the app should be used?

- ◯ Approximately Daily
- ◯ Approximately Weekly
- ◯ Approximately Monthly
- ◯ Approximately Yearly
- ◯ "as needed"
- ◉ Autre : No peculiar information

Approx. Percentage of Users (starters) still using the app as recommended after 3 months *

- ◯ unknown / not evaluated
- ◯ 0-10%
- ◯ 11-20%
- ◯ 21-30%
- ◯ 31-40%
- ◯ 41-50%
- ◯ 51-60%
- ◯ 61-70%
- ◯ 71%-80%
- ◯ 81-90%
- ◯ 91-100%
- ◉ Autre : No peculiar information

Overall, was the app/intervention effective? *

- ○ yes: all primary outcomes were significantly better in intervention group vs control
- ◉ partly: SOME primary outcomes were significantly better in intervention group vs control
- ○ no statistically significant difference between control and intervention
- ○ potentially harmful: control was significantly better than intervention in one or more outcomes
- ○ inconclusive: more research is needed
- ○ Autre :

Article Preparation Status/Stage *

At which stage in your article preparation are you currently (at the time you fill in this form)

- ○ not submitted yet - in early draft status
- ◉ not submitted yet - in late draft status, just before submission
- ○ submitted to a journal but not reviewed yet
- ○ submitted to a journal and after receiving initial reviewer comments
- ○ submitted to a journal and accepted, but not published yet
- ○ published
- ○ Autre :

Journal *

If you already know where you will submit this paper (or if it is already submitted), please provide the journal name (if it is not JMIR, provide the journal name under "other")

- ( ) not submitted yet / unclear where I will submit this
- ( ) Journal of Medical Internet Research (JMIR)
- ( ) JMIR mHealth and UHealth
- (•) JMIR Serious Games
- ( ) JMIR Mental Health
- ( ) JMIR Public Health
- ( ) JMIR Formative Research
- ( ) Other JMIR sister journal
- ( ) Autre :

Is this a full powered effectiveness trial or a pilot/feasibility trial? *

- (•) Pilot/feasibility
- ( ) Fully powered

Manuscript tracking number *

If this is a JMIR submission, please provide the manuscript tracking number under "other" (The ms tracking number can be found in the submission acknowledgement email, or when you login as author in JMIR. If the paper is already published in JMIR, then the ms tracking number is the four-digit number at the end of the DOI, to be found at the bottom of each published article in JMIR)

- (•) no ms number (yet) / not (yet) submitted to / published in JMIR
- ( ) Autre :

TITLE AND ABSTRACT

1a) TITLE: Identification as a randomized trial in the title

1a) Does your paper address CONSORT item 1a? *

I.e does the title contain the phrase "Randomized Controlled Trial"? (if not, explain the reason under "other")

- ○ yes
- ◉ Autre : Interventional study with a pretest-posttest design, an experimental g

1a-i) Identify the mode of delivery in the title

Identify the mode of delivery. Preferably use “web-based” and/or “mobile” and/or “electronic game” in the title. Avoid ambiguous terms like “online”, “virtual”, “interactive”. Use “Internet-based” only if Intervention includes non-web-based Internet components (e.g. email), use “computer-based” or “electronic” only if offline products are used. Use “virtual” only in the context of “virtual reality” (3-D worlds). Use “online” only in the context of “online support groups”. Complement or substitute product names with broader terms for the class of products (such as “mobile” or “smart phone” instead of “iphone”), especially if the application runs on different platforms.

| | 1 | 2 | 3 | 4 | 5 | |
|---|---|---|---|---|---|---|
| subitem not at all important | ○ | ○ | ◉ | ○ | ○ | essential |

Effacer la sélection

Does your paper address subitem 1a-i? *

Copy and paste relevant sections from manuscript title (include quotes in quotation marks "like this" to indicate direct quotes from your manuscript), or elaborate on this item by providing additional information not in the ms, or briefly explain why the item is not applicable/relevant for your study

"Effect of dance-based exergaming"

1a-ii) Non-web-based components or important co-interventions in title

Mention non-web-based components or important co-interventions in title, if any (e.g., “with telephone support”).

| | 1 | 2 | 3 | 4 | 5 | |
|---|---|---|---|---|---|---|
| subitem not at all important | ○ | ○ | ● | ○ | ○ | essential |

Effacer la sélection

Does your paper address subitem 1a-ii?

Copy and paste relevant sections from manuscript title (include quotes in quotation marks "like this" to indicate direct quotes from your manuscript), or elaborate on this item by providing additional information not in the ms, or briefly explain why the item is not applicable/relevant for your study

"Effect of dance-based exergaming"

1a-iii) Primary condition or target group in the title

Mention primary condition or target group in the title, if any (e.g., “for children with Type I Diabetes”) Example: A Web-based and Mobile Intervention with Telephone Support for Children with Type I Diabetes: Randomized Controlled Trial

| | 1 | 2 | 3 | 4 | 5 | |
|---|---|---|---|---|---|---|
| subitem not at all important | ○ | ○ | ○ | ○ | ● | essential |

Effacer la sélection

Does your paper address subitem 1a-iii? *

Copy and paste relevant sections from manuscript title (include quotes in quotation marks "like this" to indicate direct quotes from your manuscript), or elaborate on this item by providing additional information not in the ms, or briefly explain why the item is not applicable/relevant for your study

"Effects of dance-based exergaming in Adolescent Students"

1b) ABSTRACT: Structured summary of trial design, methods, results, and conclusions

NPT extension: Description of experimental treatment, comparator, care providers, centers, and blinding status.

1b-i) Key features/functionalities/components of the intervention and comparator in the METHODS section of the ABSTRACT

Mention key features/functionalities/components of the intervention and comparator in the abstract. If possible, also mention theories and principles used for designing the site. Keep in mind the needs of systematic reviewers and indexers by including important synonyms. (Note: Only report in the abstract what the main paper is reporting. If this information is missing from the main body of text, consider adding it)

| | 1 | 2 | 3 | 4 | 5 | |
|---|---|---|---|---|---|---|
| subitem not at all important | ○ | ○ | ○ | ○ | ◉ | essential |

Effacer la sélection

Does your paper address subitem 1b-i? *

Copy and paste relevant sections from the manuscript abstract (include quotes in quotation marks "like this" to indicate direct quotes from your manuscript), or elaborate on this item by providing additional information not in the ms, or briefly explain why the item is not applicable/relevant for your study

"EG was involved in dance-based exergaming (DEx) with different types of locomotion and interlimb coordination, and CG, in exergaming based on precision ball-throwing (TEx), requiring catching and throwing a ball. Only DEx involved mental rotation, and neither DEx nor TEx involved mathematical content. DEx and TEx were sequences of 5 weekly 45-minute sessions, implemented using the Lü platform."

## 1b-ii) Level of human involvement in the METHODS section of the ABSTRACT

Clarify the level of human involvement in the abstract, e.g., use phrases like “fully automated” vs. “therapist/nurse/care provider/physician-assisted” (mention number and expertise of providers involved, if any). (Note: Only report in the abstract what the main paper is reporting. If this information is missing from the main body of text, consider adding it)

| | 1 | 2 | 3 | 4 | 5 | |
|---|---|---|---|---|---|---|
| subitem not at all important | ○ | ○ | ○ | ○ | ◉ | essential |

Effacer la sélection

## Does your paper address subitem 1b-ii?

Copy and paste relevant sections from the manuscript abstract (include quotes in quotation marks "like this" to indicate direct quotes from your manuscript), or elaborate on this item by providing additional information not in the ms, or briefly explain why the item is not applicable/relevant for your study

"An experimental group (EG) with 15 girls and 15 boys (mean 14.0, SD 0.7 years) and a control group (CG) with 14 boys and 12 girls (mean 14.2, SD 0.9 years) participated in the study. EG was involved in dance-based exergaming (DEx) with different types of locomotion and interlimb coordination, and CG, in exergaming based on precision ball-throwing (TEx), requiring catching and throwing a ball."

1b-iii) Open vs. closed, web-based (self-assessment) vs. face-to-face assessments in the METHODS section of the ABSTRACT

Mention how participants were recruited (online vs. offline), e.g., from an open access website or from a clinic or a closed online user group (closed usergroup trial), and clarify if this was a purely web-based trial, or there were face-to-face components (as part of the intervention or for assessment). Clearly say if outcomes were self-assessed through questionnaires (as common in web-based trials). Note: In traditional offline trials, an open trial (open-label trial) is a type of clinical trial in which both the researchers and participants know which treatment is being administered. To avoid confusion, use “blinded” or “unblinded” to indicated the level of blinding instead of “open”, as “open” in web-based trials usually refers to “open access” (i.e. participants can self-enrol). (Note: Only report in the abstract what the main paper is reporting. If this information is missing from the main body of text, consider adding it)

| | 1 | 2 | 3 | 4 | 5 | |
|---|---|---|---|---|---|---|
| subitem not at all important | ○ | ○ | ○ | ○ | ◉ | essential |

Effacer la sélection

Does your paper address subitem 1b-iii?

Copy and paste relevant sections from the manuscript abstract (include quotes in quotation marks "like this" to indicate direct quotes from your manuscript), or elaborate on this item by providing additional information not in the ms, or briefly explain why the item is not applicable/relevant for your study

"A pretest-posttest design allowed to compare DEx and TEx influence on (1) a mental rotation test, (2) locomotion and sprint tests, and (3) quantity comparison tests and simple and complex addition and multiplication tests. Each session led to non-instrumented observation and sessions 3 and 5, to instrumented tracking of physical activity and situational interest."

1b-iv) RESULTS section in abstract must contain use data

Report number of participants enrolled/assessed in each group, the use/uptake of the intervention (e.g., attrition/adherence metrics, use over time, number of logins etc.), in addition to primary/secondary outcomes. (Note: Only report in the abstract what the main paper is reporting. If this information is missing from the main body of text, consider adding it)

| | 1 | 2 | 3 | 4 | 5 | |
|---|---|---|---|---|---|---|
| subitem not at all important | ○ | ○ | ○ | ○ | ◉ | essential |

Effacer la sélection

Does your paper address subitem 1b-iv?

Copy and paste relevant sections from the manuscript abstract (include quotes in quotation marks "like this" to indicate direct quotes from your manuscript), or elaborate on this item by providing additional information not in the ms, or briefly explain why the item is not applicable/relevant for your study

"An experimental group (EG) with 15 girls and 15 boys (mean 14.0, SD 0.7 years) and a control group (CG) with 14 boys and 12 girls (mean 14.2, SD 0.9 years) participated in the study. EG was involved in dance-based exergaming (DEx) with different types of locomotion and interlimb coordination, and CG, in exergaming based on precision ball-throwing (TEx), requiring catching and throwing a ball. Only DEx involved mental rotation, and neither DEx nor TEx involved mathematical content. DEx and TEx were sequences of 5 weekly 45-minute sessions, implemented using the Lü platform."
"Monitoring of the sequences showed only a significant between-group difference in terms of triggered situational interest in session 5 (MANOVA results with $F_{1,54}=9.15$, $P=.004$, $\eta p^2=.14$). The variable was added as a covariate in the ANCOVAs with GLM approach performed further. ANCOVA results showed an advantage for EG over CG in terms of mental rotation ($F_{1,52}=6.17$, $P=.016$, $\eta p^2=.11$), number of correct simple additions ($F_{1,52}=8.26$, $P=.006$, $\eta p^2=.14$), and error rate in complex addition ($F_{1,52}=8.40$, $P=.005$, $\eta p^2=.14$). No other significant results were found; locomotor performance improved in EG (by 3%) and CG (by 5%) from pretest to posttest."

### 1b-v) CONCLUSIONS/DISCUSSION in abstract for negative trials

Conclusions/Discussions in abstract for negative trials: Discuss the primary outcome - if the trial is negative (primary outcome not changed), and the intervention was not used, discuss whether negative results are attributable to lack of uptake and discuss reasons. (Note: Only report in the abstract what the main paper is reporting. If this information is missing from the main body of text, consider adding it)

| | 1 | 2 | 3 | 4 | 5 | |
|---|---|---|---|---|---|---|
| subitem not at all important | ○ | ○ | ○ | ◉ | ○ | essential |

Effacer la sélection

### Does your paper address subitem 1b-v?

Copy and paste relevant sections from the manuscript abstract (include quotes in quotation marks "like this" to indicate direct quotes from your manuscript), or elaborate on this item by providing additional information not in the ms, or briefly explain why the item is not applicable/relevant for your study

"DEx improved mental rotation efficiency and only calculations involving mental rotation according to the literature. Further research is needed to clarify whether such improvement in calculation is linked to mental rotation training itself and whether training general motor coordination might influence this improvement."

## INTRODUCTION

### 2a) In INTRODUCTION: Scientific background and explanation of rationale

2a-i) Problem and the type of system/solution

Describe the problem and the type of system/solution that is object of the study: intended as stand-alone intervention vs. incorporated in broader health care program? Intended for a particular patient population? Goals of the intervention, e.g., being more cost-effective to other interventions, replace or complement other solutions? (Note: Details about the intervention are provided in “Methods” under 5)

| | 1 | 2 | 3 | 4 | 5 | |
|---|---|---|---|---|---|---|
| subitem not at all important | ○ | ○ | ○ | ○ | ◉ | essential |

Effacer la sélection

Does your paper address subitem 2a-i? *

Copy and paste relevant sections from the manuscript (include quotes in quotation marks "like this" to indicate direct quotes from your manuscript), or elaborate on this item by providing additional information not in the ms, or briefly explain why the item is not applicable/relevant for your study

"Based on the literature, our study aimed to determine whether practicing a dance-based exergame, with no overt math content, might positively influence mental rotation efficiency, general motor coordination, and math achievement in adolescent students."
The specific features of this study, compared to previous studies on the possible influence of active video games on academic performance, are described in more detail in the manuscript.

2a-ii) Scientific background, rationale: What is known about the (type of) system

Scientific background, rationale: What is known about the (type of) system that is the object of the study (be sure to discuss the use of similar systems for other conditions/diagnoses, if appropiate), motivation for the study, i.e. what are the reasons for and what is the context for this specific study, from which stakeholder viewpoint is the study performed, potential impact of findings [2]. Briefly justify the choice of the comparator.

| | 1 | 2 | 3 | 4 | 5 | |
|---|---|---|---|---|---|---|
| subitem not at all important | ○ | ○ | ○ | ○ | ◉ | essential |

Effacer la sélection

Does your paper address subitem 2a-ii? *

Copy and paste relevant sections from the manuscript (include quotes in quotation marks "like this" to indicate direct quotes from your manuscript), or elaborate on this item by providing additional information not in the ms, or briefly explain why the item is not applicable/relevant for your study

"From the outset, authors have argued that exergames may influence health by favoring physical training and a positive attitude towards physical activity [9]."
"On the other hand, the attractiveness of practicing an exergame might favor the repetition of the tasks to be performed when exergaming, thus learning these tasks, as authors have long considered repetition to be a key element of learning [21,22]..
Most studies examined such possible influence of exergaming on motor learning outside the school context, and especially fundamental motor skills learning. These [...] fform a basis for learning and performing different types of physical activity [23-25]. [...] Especially, whether exergaming might develop general motor coordination (i.e., the ability to coordinate several limbs and the whole body [31] as a basis for locomotor skills [23]) remains, to our knowledge, to be clarified.
Furthermore, the influence of exergaming on school achievement has been little studied to date, although studies emphasized that active learning, which links movement and learning, may be a promising way to reduce sedentary behavior while improving academic performance [32,33]. [...] The last result thus suggests that practicing an exergame might positively influence school achievement beyond the area(s) that this exergame targets, which requires further investigation as such a possibility would be of great pedagogical interest. [...] Another explanation might lie in the development, thanks to exergaming, of spatial ability, that is, a component of intellectual ability [41] and a predictor of math achievement [42]. [...] IInterestingly, studies found that the practice of physical activities in which the processing of spatial information is decisive (e.g., wrestling [47] and dance [48]) may improve mental rotation, that is, a component of spatial ability that allows one to rotate the mental image of an object [45,46]. Other studies showed that mental rotation training may positively influence math achievement (e.g., [49]). More results suggested the existence of links among mental rotation, general motor coordination, and math achievement, particularly in arithmetic [50-52]."

2b) In INTRODUCTION: Specific objectives or hypotheses

Does your paper address CONSORT subitem 2b? *

Copy and paste relevant sections from the manuscript (include quotes in quotation marks "like this" to indicate direct quotes from your manuscript), or elaborate on this item by providing additional information not in the ms, or briefly explain why the item is not applicable/relevant for your study

"The main objective of this study was to determine the influence of this practice on mental rotation efficiency, general motor coordination, and math achievement in an experimental group (EG). This was done by comparison with a control group (CG) involved in exergaming based on precision ball throwing, using a pretest-posttest design. Students' intensity levels of physical activity during exergaming and the situational interest [53] that this practice generated in them were also measured to monitor their involvement."

METHODS

3a) Description of trial design (such as parallel, factorial) including allocation ratio

Does your paper address CONSORT subitem 3a? *

Copy and paste relevant sections from the manuscript (include quotes in quotation marks "like this" to indicate direct quotes from your manuscript), or elaborate on this item by providing additional information not in the ms, or briefly explain why the item is not applicable/relevant for your study

"The sequences of exergaming, based on dance (DEx) in EG and precision ball-throwing (TEx) in CG, were implemented for five weeks during the regular PE timetable of the classes involved in the experiment. The participants attended two weekly PE lessons: one 90-minute lesson (“two-period lesson”) and one 45-minute lesson (“one-period lesson”). Only the first period of each two-period lesson differed between EG and CG, with DEx sessions in EG and TEx sessions in CG. [...] Both EG and CG practiced badminton during the second period of each two-period lesson and volleyball during each one-period lesson (Figure 2). The same experimenters observed each session of DEx and TEx to check whether the teacher had implemented it as planned. The intensity levels of the EG and CG subjects’ physical activity were also measured to check whether these were in line with the respective exergaming demands (Figure 2). This also allowed an estimation of the exergaming contribution to the moderate-to-vigorous physical activity (MVPA) recommended daily by the World Health Organization [54]. Measurements were done during sessions 3 and 5. The experimenters monitored physical activity, as in previous studies (e.g., [55]), during the sessions at the end of which situational interest was assessed to control for possible differences between EG and CG likely to have influenced activity during exergaming (Figure 2). [...]
The students completed pretests during the week preceding the beginning of DEx and TEx and posttests during the week posterior to DEx and TEx. During both pretests and posttests, EG and CG performed a mental rotation test, a sprint race and a locomotion circuit used to assess general motor coordination, and math tests. EG and CG subjects performed mental rotation tests and math tests at the beginning of two separate math lessons. These subjects also performed the sprint race during the one-period PE lesson, and the locomotion circuit, during the two-period PE lesson. The experimenters also asked science and math teachers in classes of the EG and CG subjects not to provide any specific training on mental rotation and the mathematical content covered in the pretests and posttests during the experiment."

3b) Important changes to methods after trial commencement (such as eligibility criteria), with reasons

Does your paper address CONSORT subitem 3b? *

Copy and paste relevant sections from the manuscript (include quotes in quotation marks "like this" to indicate direct quotes from your manuscript), or elaborate on this item by providing additional information not in the ms, or briefly explain why the item is not applicable/relevant for your study

No changes to methods after trial commencement.

3b-i) Bug fixes, Downtimes, Content Changes

Bug fixes, Downtimes, Content Changes: ehealth systems are often dynamic systems. A description of changes to methods therefore also includes important changes made on the intervention or comparator during the trial (e.g., major bug fixes or changes in the functionality or content) (5-iii) and other “unexpected events” that may have influenced study design such as staff changes, system failures/downtimes, etc. [2].

| | 1 | 2 | 3 | 4 | 5 | |
|---|---|---|---|---|---|---|
| subitem not at all important | ○ | ○ | ○ | ○ | ○ | essential |

Does your paper address subitem 3b-i?

Copy and paste relevant sections from the manuscript (include quotes in quotation marks "like this" to indicate direct quotes from your manuscript), or elaborate on this item by providing additional information not in the ms, or briefly explain why the item is not applicable/relevant for your study

Not relevant in this study.

4a) Eligibility criteria for participants

Does your paper address CONSORT subitem 4a? *

Copy and paste relevant sections from the manuscript (include quotes in quotation marks "like this" to indicate direct quotes from your manuscript), or elaborate on this item by providing additional information not in the ms, or briefly explain why the item is not applicable/relevant for your study

"This study focused on two groups, EG and CG, composed of 13-16-year-old students from a secondary school based in Switzerland (canton of Vaud). Figure 1 shows the different stages involved in setting up the groups."
"Two qualified PE teachers, with experience in teaching (10 years) and familiarity with their school's Lü exergaming system, participated in the study. One was the regular PE teacher for the two classes composing EG, and the other for the two classes composing CG."
"We presented the project to the school's headteacher, who agreed to its implementation. The teachers involved in the experiment, the parents of the students, and the students completed written informed consent gathered prior to data collection, which gave them the option to withdraw their consent at any time."

4a-i) Computer / Internet literacy

Computer / Internet literacy is often an implicit "de facto" eligibility criterion - this should be explicitly clarified.

| | 1 | 2 | 3 | 4 | 5 | |
|---|---|---|---|---|---|---|
| subitem not at all important | ○ | ○ | ○ | ○ | ○ | essential |

Does your paper address subitem 4a-i?

Copy and paste relevant sections from the manuscript (include quotes in quotation marks "like this" to indicate direct quotes from your manuscript), or elaborate on this item by providing additional information not in the ms, or briefly explain why the item is not applicable/relevant for your study

Not relevant in this study.

4a-ii) Open vs. closed, web-based vs. face-to-face assessments:

Open vs. closed, web-based vs. face-to-face assessments: Mention how participants were recruited (online vs. offline), e.g., from an open access website or from a clinic, and clarify if this was a purely web-based trial, or there were face-to-face components (as part of the intervention or for assessment), i.e., to what degree got the study team to know the participant. In online-only trials, clarify if participants were quasi-anonymous and whether having multiple identities was possible or whether technical or logistical measures (e.g., cookies, email confirmation, phone calls) were used to detect/prevent these.

| | 1 | 2 | 3 | 4 | 5 | |
|---|---|---|---|---|---|---|
| subitem not at all important | ○ | ○ | ○ | ● | ○ | essential |

Effacer la sélection

Does your paper address subitem 4a-ii? *

Copy and paste relevant sections from the manuscript (include quotes in quotation marks "like this" to indicate direct quotes from your manuscript), or elaborate on this item by providing additional information not in the ms, or briefly explain why the item is not applicable/relevant for your study

"We constituted EG from two classes of the school where the experiment took place, and CG, from two other classes of this school."
"The experimenters monitored physical activity, as in previous studies (e.g., [55]), during the sessions at the end of which situational interest was assessed to control for possible differences between EG and CG likely to have influenced activity during exergaming (Figure 2). These sessions were chosen to avoid novelty bias (session 3) and allow time for possible evolution of situational interest (session 5, i.e., the last of DEx and TEx), as in previous studies (e.g., [35])."

### 4a-iii) Information giving during recruitment

Information given during recruitment. Specify how participants were briefed for recruitment and in the informed consent procedures (e.g., publish the informed consent documentation as appendix, see also item X26), as this information may have an effect on user self-selection, user expectation and may also bias results.

| | 1 | 2 | 3 | 4 | 5 | |
|---|---|---|---|---|---|---|
| subitem not at all important | ○ | ○ | ○ | ○ | ◉ | essential |

Effacer la sélection

### Does your paper address subitem 4a-iii?

Copy and paste relevant sections from the manuscript (include quotes in quotation marks "like this" to indicate direct quotes from your manuscript), or elaborate on this item by providing additional information not in the ms, or briefly explain why the item is not applicable/relevant for your study

"We presented the project to the school's headteacher, who agreed to its implementation. The teachers involved in the experiment, the parents of the students, and the students completed written informed consent gathered prior to data collection, which gave them the option to withdraw their consent at any time."

### 4b) Settings and locations where the data were collected

Does your paper address CONSORT subitem 4b? *

Copy and paste relevant sections from the manuscript (include quotes in quotation marks "like this" to indicate direct quotes from your manuscript), or elaborate on this item by providing additional information not in the ms, or briefly explain why the item is not applicable/relevant for your study

"This study focused on two groups, EG and CG, composed of 13-16-year-old students from a secondary school based in Switzerland (canton of Vaud)."
"The sequences of exergaming, based on dance (DEx) in EG and precision ball-throwing (TEx) in CG, were implemented for five weeks during the regular PE timetable of the classes involved in the experiment. The participants attended two weekly PE lessons: one 90-minute lesson ("two-period lesson") and one 45-minute lesson ("one-period lesson"). Only the first period of each two-period lesson differed between EG and CG, with DEx sessions in EG and TEx sessions in CG."
"The same experimenters observed each session of DEx and TEx to check whether the teacher had implemented it as planned. The intensity levels of the EG and CG subjects' physical activity were also measured to check whether these were in line with the respective exergaming demands (Figure 2)."
"The experimenters monitored physical activity, as in previous studies (e.g., [55]), during the sessions at the end of which situational interest was assessed to control for possible differences between EG and CG likely to have influenced activity during exergaming (Figure 2)."
"The students completed pretests during the week preceding the beginning of DEx and TEx and posttests during the week posterior to DEx and TEx. During both pretests and posttests, EG and CG performed a mental rotation test, a sprint race and a locomotion circuit used to assess general motor coordination, and math tests. EG and CG subjects performed mental rotation tests and math tests at the beginning of two separate math lessons. These subjects also performed the sprint race during the one-period PE lesson, and the locomotion circuit, during the two-period PE lesson."

4b-i) Report if outcomes were (self-)assessed through online questionnaires

Clearly report if outcomes were (self-)assessed through online questionnaires (as common in web-based trials) or otherwise.

| | 1 | 2 | 3 | 4 | 5 | |
|---|---|---|---|---|---|---|
| subitem not at all important | ○ | ○ | ○ | ○ | ○ | essential |

Does your paper address subitem 4b-i? *

Copy and paste relevant sections from the manuscript (include quotes in quotation marks "like this" to indicate direct quotes from your manuscript), or elaborate on this item by providing additional information not in the ms, or briefly explain why the item is not applicable/relevant for your study

Not relevant in this study.

4b-ii) Report how institutional affiliations are displayed

Report how institutional affiliations are displayed to potential participants [on ehealth media], as affiliations with prestigious hospitals or universities may affect volunteer rates, use, and reactions with regards to an intervention.(Not a required item – describe only if this may bias results)

| | 1 | 2 | 3 | 4 | 5 | |
|---|---|---|---|---|---|---|
| subitem not at all important | ○ | ○ | ◉ | ○ | ○ | essential |

Effacer la sélection

Does your paper address subitem 4b-ii?

Copy and paste relevant sections from the manuscript (include quotes in quotation marks "like this" to indicate direct quotes from your manuscript), or elaborate on this item by providing additional information not in the ms, or briefly explain why the item is not applicable/relevant for your study

"In accordance with local procedure, the project for this pilot interventional study was submitted to the Research Coordination Committee of the host university, based in Switzerland (canton of Vaud). This committee approved the project without requiring further submission to the Cantonal Commission for Ethics in Human Research. Subsequently, this project has been integrated into the university's Research Project Repository.
We also obtained official authorization to implement the study in schools located in the canton of Vaud and selected one secondary school for the study due to its exergaming facilities. We presented the project to the school's headteacher, who agreed to its implementation."

5) The interventions for each group with sufficient details to allow replication, including how and when they were actually administered

5-i) Mention names, credential, affiliations of the developers, sponsors, and owners

Mention names, credential, affiliations of the developers, sponsors, and owners [6] (if authors/evaluators are owners or developer of the software, this needs to be declared in a “Conflict of interest” section or mentioned elsewhere in the manuscript).

| | 1 | 2 | 3 | 4 | 5 | |
|---|---|---|---|---|---|---|
| subitem not at all important | ○ | ○ | ○ | ○ | ◉ | essential |

Effacer la sélection

Does your paper address subitem 5-i?

Copy and paste relevant sections from the manuscript (include quotes in quotation marks "like this" to indicate direct quotes from your manuscript), or elaborate on this item by providing additional information not in the ms, or briefly explain why the item is not applicable/relevant for your study

"The teachers participating in the study implemented DEx and TEx using a Lü Üno system, that is, an audiovisual infrastructure devoted to exergames practice and coming with gaming applications."

5-ii) Describe the history/development process

Describe the history/development process of the application and previous formative evaluations (e.g., focus groups, usability testing), as these will have an impact on adoption/use rates and help with interpreting results.

| | 1 | 2 | 3 | 4 | 5 | |
|---|---|---|---|---|---|---|
| subitem not at all important | ○ | ○ | ○ | ◉ | ○ | essential |

Effacer la sélection

Does your paper address subitem 5-ii?

Copy and paste relevant sections from the manuscript (include quotes in quotation marks "like this" to indicate direct quotes from your manuscript), or elaborate on this item by providing additional information not in the ms, or briefly explain why the item is not applicable/relevant for your study

"Two qualified PE teachers, with experience in teaching (10 years) and familiarity with their school's Lü exergaming system, participated in the study."
"DEx and TEx sessions have been designed separately, each by a group including the researchers and the PE teacher who would teach the sequence (either to EG, or to CG). Prior to the experiment, each sequence was tested with other students than those forming EG and CG. This enabled the teachers involved in the study to perfect their use of the Lü Üno system, as DEx and TEx included elements (content, practice options) that they needed to explore further. This also led to adjusting the sequences for subsequent implementation."

5-iii) Revisions and updating

Revisions and updating. Clearly mention the date and/or version number of the application/intervention (and comparator, if applicable) evaluated, or describe whether the intervention underwent major changes during the evaluation process, or whether the development and/or content was "frozen" during the trial. Describe dynamic components such as news feeds or changing content which may have an impact on the replicability of the intervention (for unexpected events see item 3b).

| | 1 | 2 | 3 | 4 | 5 | |
|---|---|---|---|---|---|---|
| subitem not at all important | ○ | ○ | ○ | ○ | ○ | essential |

Does your paper address subitem 5-iii?

Copy and paste relevant sections from the manuscript (include quotes in quotation marks "like this" to indicate direct quotes from your manuscript), or elaborate on this item by providing additional information not in the ms, or briefly explain why the item is not applicable/relevant for your study

The intervention was aimed to be done as planned.

5-iv) Quality assurance methods

Provide information on quality assurance methods to ensure accuracy and quality of information provided [1], if applicable.

| | 1 | 2 | 3 | 4 | 5 | |
|---|---|---|---|---|---|---|
| subitem not at all important | ○ | ○ | ○ | ◉ | ○ | essential |

Effacer la sélection

Does your paper address subitem 5-iv?

Copy and paste relevant sections from the manuscript (include quotes in quotation marks "like this" to indicate direct quotes from your manuscript), or elaborate on this item by providing additional information not in the ms, or briefly explain why the item is not applicable/relevant for your study

"The same experimenters observed each session of DEx and TEx to check whether the teacher had implemented it as planned. The intensity levels of the EG and CG subjects' physical activity were also measured to check whether these were in line with the respective exergaming demands (Figure 2)."
"The experimenters monitored physical activity, as in previous studies (e.g., [55]), during the sessions at the end of which situational interest was assessed to control for possible differences between EG and CG likely to have influenced activity during exergaming (Figure 2). These sessions were chosen to avoid novelty bias (session 3) and allow time for possible evolution of situational interest (session 5, i.e., the last of DEx and TEx), as in previous studies (e.g., [35])."

5-v) Ensure replicability by publishing the source code, and/or providing screenshots/screen-capture video, and/or providing flowcharts of the algorithms used

Ensure replicability by publishing the source code, and/or providing screenshots/screen-capture video, and/or providing flowcharts of the algorithms used. Replicability (i.e., other researchers should in principle be able to replicate the study) is a hallmark of scientific reporting.

| | 1 | 2 | 3 | 4 | 5 | |
|---|---|---|---|---|---|---|
| subitem not at all important | ○ | ○ | ○ | ○ | ○ | essential |

Does your paper address subitem 5-v?

Copy and paste relevant sections from the manuscript (include quotes in quotation marks "like this" to indicate direct quotes from your manuscript), or elaborate on this item by providing additional information not in the ms, or briefly explain why the item is not applicable/relevant for your study

Not relevant in this study.

5-vi) Digital preservation

Digital preservation: Provide the URL of the application, but as the intervention is likely to change or disappear over the course of the years; also make sure the intervention is archived (Internet Archive, webcitation.org, and/or publishing the source code or screenshots/videos alongside the article). As pages behind login screens cannot be archived, consider creating demo pages which are accessible without login.

| | 1 | 2 | 3 | 4 | 5 | |
|---|---|---|---|---|---|---|
| subitem not at all important | ○ | ○ | ○ | ○ | ○ | essential |

Does your paper address subitem 5-vi?

Copy and paste relevant sections from the manuscript (include quotes in quotation marks "like this" to indicate direct quotes from your manuscript), or elaborate on this item by providing additional information not in the ms, or briefly explain why the item is not applicable/relevant for your study

Not relevant in this study.

5-vii) Access

Access: Describe how participants accessed the application, in what setting/context, if they had to pay (or were paid) or not, whether they had to be a member of specific group. If known, describe how participants obtained “access to the platform and Internet” [1]. To ensure access for editors/reviewers/readers, consider to provide a “backdoor” login account or demo mode for reviewers/readers to explore the application (also important for archiving purposes, see vi).

| | 1 | 2 | 3 | 4 | 5 | |
|---|---|---|---|---|---|---|
| subitem not at all important | ○ | ○ | ○ | ◉ | ○ | essential |

Effacer la sélection

Does your paper address subitem 5-vii? *

Copy and paste relevant sections from the manuscript (include quotes in quotation marks "like this" to indicate direct quotes from your manuscript), or elaborate on this item by providing additional information not in the ms, or briefly explain why the item is not applicable/relevant for your study

"We [...] selected one secondary school for the study due to its exergaming facilities."
"The sequences of exergaming, based on dance (DEx) in EG and precision ball-throwing (TEx) in CG, were implemented for five weeks during the regular PE timetable of the classes involved in the experiment."

5-viii) Mode of delivery, features/functionalities/components of the intervention and comparator, and the theoretical framework

Describe mode of delivery, features/functionalities/components of the intervention and comparator, and the theoretical framework [6] used to design them (instructional strategy [1], behaviour change techniques, persuasive features, etc., see e.g., [7, 8] for terminology). This includes an in-depth description of the content (including where it is coming from and who developed it) [1]," whether [and how] it is tailored to individual circumstances and allows users to track their progress and receive feedback" [6]. This also includes a description of communication delivery channels and – if computer-mediated communication is a component – whether communication was synchronous or asynchronous [6]. It also includes information on presentation strategies [1], including page design principles, average amount of text on pages, presence of hyperlinks to other resources, etc. [1].

| | 1 | 2 | 3 | 4 | 5 | |
|---|---|---|---|---|---|---|
| subitem not at all important | ○ | ○ | ○ | ○ | ◉ | essential |

Effacer la sélection

Does your paper address subitem 5-viii? *

Copy and paste relevant sections from the manuscript (include quotes in quotation marks "like this" to indicate direct quotes from your manuscript), or elaborate on this item by providing additional information not in the ms, or briefly explain why the item is not applicable/relevant for your study

"The sequences of exergaming, based on dance (DEx) in EG and precision ball-throwing (TEx) in CG, were implemented for five weeks during the regular PE timetable of the classes involved in the experiment. The participants attended two weekly PE lessons: one 90-minute lesson ("two-period lesson") and one 45-minute lesson ("one-period lesson"). Only the first period of each two-period lesson differed between EG and CG, with DEx sessions in EG and TEx sessions in CG. No student declared previous experience of the exergames practiced during these sequences. Both EG and CG practiced badminton during the second period of each two-period lesson and volleyball during each one-period lesson (Figure 2)."

5-ix) Describe use parameters

Describe use parameters (e.g., intended "doses" and optimal timing for use). Clarify what instructions or recommendations were given to the user, e.g., regarding timing, frequency, heaviness of use, if any, or was the intervention used ad libitum.

| | 1 | 2 | 3 | 4 | 5 | |
|---|---|---|---|---|---|---|
| subitem not at all important | ○ | ○ | ○ | ○ | ◉ | essential |

Effacer la sélection

Does your paper address subitem 5-ix?

Copy and paste relevant sections from the manuscript (include quotes in quotation marks "like this" to indicate direct quotes from your manuscript), or elaborate on this item by providing additional information not in the ms, or briefly explain why the item is not applicable/relevant for your study

"The sequences of exergaming, based on dance (DEx) in EG and precision ball-throwing (TEx) in CG, were implemented for five weeks during the regular PE timetable of the classes involved in the experiment."
"EG practiced Grööve with the explicit aim of learning a dance based on a song including four verses and a chorus."
"Thus, to maximize the use of mental rotation, the teacher had to instruct the EG subjects to imagine themselves dancing in the position of one of the avatars facing them, that is, to rotate mentally their own body to get into this position. The teacher had to give this instruction before each dancing phase, to reiterate it during learning, and when providing feedback. Each session, the teacher had also to film the dance movements of the EG subjects, with the Lü camera used facing them (Figure 3). At the end of the session, the teacher had to use the videos for formative evaluation and to train the subjects to analyze freeze-frames with the same instruction as when imitating an avatar (e.g., to determine which arm of the viewed subject was outstretched)."
"The teacher implemented TEx using eight Lü applications, each leading to throwing a ball at a target, in addition to catching it and performing simple walk or run locomotion (see: Multimedia Appendix 2)."

### 5-x) Clarify the level of human involvement

Clarify the level of human involvement (care providers or health professionals, also technical assistance) in the e-intervention or as co-intervention (detail number and expertise of professionals involved, if any, as well as “type of assistance offered, the timing and frequency of the support, how it is initiated, and the medium by which the assistance is delivered”. It may be necessary to distinguish between the level of human involvement required for the trial, and the level of human involvement required for a routine application outside of a RCT setting (discuss under item 21 – generalizability).

| | 1 | 2 | 3 | 4 | 5 | |
|---|---|---|---|---|---|---|
| subitem not at all important | ○ | ○ | ○ | ● | ○ | essential |

Effacer la sélection

### Does your paper address subitem 5-x?

Copy and paste relevant sections from the manuscript (include quotes in quotation marks "like this" to indicate direct quotes from your manuscript), or elaborate on this item by providing additional information not in the ms, or briefly explain why the item is not applicable/relevant for your study

"Two qualified PE teachers, with experience in teaching (10 years) and familiarity with their school’s Lü exergaming system, participated in the study. One was the regular PE teacher for the two classes composing EG, and the other for the two classes composing CG."
"DEx and TEx sessions have been designed separately, each by a group including the researchers and the PE teacher who would teach the sequence (either to EG, or to CG). Prior to the experiment, each sequence was tested with other students than those forming EG and CG. This enabled the teachers involved in the study to perfect their use of the Lü Üno system, as DEx and TEx included elements (content, practice options) that they needed to explore further. This also led to adjusting the sequences for subsequent implementation."

5-xi) Report any prompts/reminders used

Report any prompts/reminders used: Clarify if there were prompts (letters, emails, phone calls, SMS) to use the application, what triggered them, frequency etc. It may be necessary to distinguish between the level of prompts/reminders required for the trial, and the level of prompts/reminders for a routine application outside of a RCT setting (discuss under item 21 – generalizability).

| | 1 | 2 | 3 | 4 | 5 | |
|---|---|---|---|---|---|---|
| subitem not at all important | ○ | ○ | ○ | ○ | ○ | essential |

Does your paper address subitem 5-xi? *

Copy and paste relevant sections from the manuscript (include quotes in quotation marks "like this"  to indicate direct quotes from your manuscript), or elaborate on this item by providing additional information not in the ms, or briefly explain why the item is not applicable/relevant for your study

Not relevant in this study.

5-xii) Describe any co-interventions (incl. training/support)

Describe any co-interventions (incl. training/support): Clearly state any interventions that are provided in addition to the targeted eHealth intervention, as ehealth intervention may not be designed as stand-alone intervention. This includes training sessions and support [1]. It may be necessary to distinguish between the level of training required for the trial, and the level of training for a routine application outside of a RCT setting (discuss under item 21 – generalizability.

| | 1 | 2 | 3 | 4 | 5 | |
|---|---|---|---|---|---|---|
| subitem not at all important | ○ | ○ | ○ | ○ | ○ | essential |

Does your paper address subitem 5-xii? *

Copy and paste relevant sections from the manuscript (include quotes in quotation marks "like this" to indicate direct quotes from your manuscript), or elaborate on this item by providing additional information not in the ms, or briefly explain why the item is not applicable/relevant for your study

Not relevant in this study.

6a) Completely defined pre-specified primary and secondary outcome measures, including how and when they were assessed

Does your paper address CONSORT subitem 6a? *

Copy and paste relevant sections from the manuscript (include quotes in quotation marks "like this" to indicate direct quotes from your manuscript), or elaborate on this item by providing additional information not in the ms, or briefly explain why the item is not applicable/relevant for your study

"For each DEx and TEx session implemented, the experimenters conducted a non-instrumented observation to enable comparison between the observed session and the planned session [...].
During sessions 3 and 5 of DEx and TEx, the experimenters also measured the intensity levels of physical activity achieved by each subject using hip-worn GT3X+ accelerometers (Actigraph Co.; Pensacola, FL, USA) [...] [57]. The accelerometer data were processed using ActiLife6. [...] The examined variables were (1) the moderate and vigorous intensity levels, expressed in seconds and percentage of time activity, and (2) MVPA, expressed similarly to (1). [...]
Directly after sessions 3 and 5, the experimenters assessed the situational interest of each subject using the French situational interest questionnaire [58] that contains three factors: (1) triggered situational interest [...], (2) maintained situational interest feeling [...], and (3) maintained situational interest value [...]. To complete the questionnaire, the subjects had to rate statements on a five-point Likert scale (see: Multimedia Appendix 3); for each factor, this led to scores ranging from 1 (lowest possible score) to 5 (highest possible score)."
"These subjects also performed a locomotion circuit called the Harre circuit test in the literature (e.g., [61]) to assess general motor coordination [62], [...] (see: Multimedia Appendix 4). In line with previous studies [52,63], a sprint race (20 meters) was performed separately to control for the influence of sprinting capacities on the circuit performance. The performance of the locomotion circuit and of the sprint race were timed using the same timing system (Race link Racing System; Brower Timing Systems, Draper, UT, USA). Moreover, the subjects performed two separate paper-and-pencil series of quantity comparisons, based on a previous study [64] (see: Multimedia Appendix 4), that is, a series of comparisons between two sets of points and a series of comparisons between two single-digit numbers. The subjects also performed four separate, paper-and-pencil, series of mental calculations, based on a previous study [65] (see: Multimedia Appendix 4), that is, series of simple additions (two single-digit numbers), complex additions (three two-digit numbers), simple multiplication (two single-digit numbers), and complex multiplication (a two-digit number and a single-digit number). For each task series listed above, we considered the total number of processed items, the number of correct results, and the error rate."

6a-i) Online questionnaires: describe if they were validated for online use and apply CHERRIES items to describe how the questionnaires were designed/deployed

If outcomes were obtained through online questionnaires, describe if they were validated for online use and apply CHERRIES items to describe how the questionnaires were designed/deployed [9].

| | 1 | 2 | 3 | 4 | 5 | |
|---|---|---|---|---|---|---|
| subitem not at all important | ○ | ○ | ○ | ○ | ○ | essential |

Does your paper address subitem 6a-i?

Copy and paste relevant sections from manuscript text

No online questionnaire was used in this study.

6a-ii) Describe whether and how “use” (including intensity of use/dosage) was defined/measured/monitored

Describe whether and how “use” (including intensity of use/dosage) was defined/measured/monitored (logins, logfile analysis, etc.). Use/adoption metrics are important process outcomes that should be reported in any ehealth trial.

| | 1 | 2 | 3 | 4 | 5 | |
|---|---|---|---|---|---|---|
| subitem not at all important | ○ | ○ | ○ | ○ | ◉ | essential |

Effacer la sélection

Does your paper address subitem 6a-ii?

Copy and paste relevant sections from manuscript text

"For each DEx and TEx session implemented, the experimenters conducted a non-instrumented observation to enable comparison between the observed session and the planned session regarding content, teacher intervention, and subject activity."
"During sessions 3 and 5 of DEx and TEx, the experimenters also measured the intensity levels of physical activity achieved by each subject using hip-worn GT3X+ accelerometers (Actigraph Co.; Pensacola, FL, USA), providing a receivable estimation of physical activity in free-living conditions [57]. The accelerometer data were processed using ActiLife6. [...]
The examined variables were (1) the moderate and vigorous intensity levels, expressed in seconds and percentage of time activity, and (2) MVPA, expressed similarly to (1). Given the exergaming characteristics in DEx and TEx described above, we expected EG to be more involved than CG in vigorous-intensity activity, and CG more involved than EG in moderate-intensity activity, as the musical tempo in DEx was to be increased during session 3 and remain at peak in session 5, whereas no such activity peaks were planned in TEx.
Directly after sessions 3 and 5, the experimenters assessed the situational interest of each subject using the French situational interest questionnaire [58] that contains three factors: (1) triggered situational interest (participant's perception of the game demanding full attention), (2) maintained situational interest feeling (to what extent the activities were found enjoyable), and (3) maintained situational interest value (importance allocated to success during the game). To complete the questionnaire, the subjects had to rate statements on a five-point Likert scale (see: Multimedia Appendix 3); for each factor, this led to scores ranging from 1 (lowest possible score) to 5 (highest possible score)."

6a-iii) Describe whether, how, and when qualitative feedback from participants was obtained

Describe whether, how, and when qualitative feedback from participants was obtained (e.g., through emails, feedback forms, interviews, focus groups).

| | 1 | 2 | 3 | 4 | 5 | |
|---|---|---|---|---|---|---|
| subitem not at all important | ○ | ○ | ○ | ○ | ○ | essential |

Does your paper address subitem 6a-iii?

Copy and paste relevant sections from manuscript text

Not relevant in this study.

6b) Any changes to trial outcomes after the trial commenced, with reasons

Does your paper address CONSORT subitem 6b? *

Copy and paste relevant sections from the manuscript (include quotes in quotation marks "like this" to indicate direct quotes from your manuscript), or elaborate on this item by providing additional information not in the ms, or briefly explain why the item is not applicable/relevant for your study

Not relevant in this study.

7a) How sample size was determined

NPT: When applicable, details of whether and how the clustering by care provides or centers was addressed

7a-i) Describe whether and how expected attrition was taken into account when calculating the sample size

Describe whether and how expected attrition was taken into account when calculating the sample size.

| | 1 | 2 | 3 | 4 | 5 | |
|---|---|---|---|---|---|---|
| subitem not at all important | ○ | ○ | ○ | ◉ | ○ | essential |

Effacer la sélection

Does your paper address subitem 7a-i?

Copy and paste relevant sections from manuscript title (include quotes in quotation marks "like this" to indicate direct quotes from your manuscript), or elaborate on this item by providing additional information not in the ms, or briefly explain why the item is not applicable/relevant for your study

"Based on preliminary data, a sample size calculation, using the “pwr" package in R, indicated that 21 participants per group would be required to achieve 90% power (α=.05, effect size of 0.5) to detect differences in the primary outcomes of the study."

7b) When applicable, explanation of any interim analyses and stopping guidelines

Does your paper address CONSORT subitem 7b? *

Copy and paste relevant sections from the manuscript (include quotes in quotation marks "like this" to indicate direct quotes from your manuscript), or elaborate on this item by providing additional information not in the ms, or briefly explain why the item is not applicable/relevant for your study

Not relevant in this study.

8a) Method used to generate the random allocation sequence
NPT: When applicable, how care providers were allocated to each trial group

Does your paper address CONSORT subitem 8a? *

Copy and paste relevant sections from the manuscript (include quotes in quotation marks "like this" to indicate direct quotes from your manuscript), or elaborate on this item by providing additional information not in the ms, or briefly explain why the item is not applicable/relevant for your study

Not relevant in this study.

8b) Type of randomisation; details of any restriction (such as blocking and block size)

Does your paper address CONSORT subitem 8b? *

Copy and paste relevant sections from the manuscript (include quotes in quotation marks "like this" to indicate direct quotes from your manuscript), or elaborate on this item by providing additional information not in the ms, or briefly explain why the item is not applicable/relevant for your study

Not relevant in this study.

9) Mechanism used to implement the random allocation sequence (such as sequentially numbered containers), describing any steps taken to conceal the sequence until interventions were assigned

Does your paper address CONSORT subitem 9? *

Copy and paste relevant sections from the manuscript (include quotes in quotation marks "like this" to indicate direct quotes from your manuscript), or elaborate on this item by providing additional information not in the ms, or briefly explain why the item is not applicable/relevant for your study

Not relevant in this study.

10) Who generated the random allocation sequence, who enrolled participants, and who assigned participants to interventions

Does your paper address CONSORT subitem 10? *

Copy and paste relevant sections from the manuscript (include quotes in quotation marks "like this" to indicate direct quotes from your manuscript), or elaborate on this item by providing additional information not in the ms, or briefly explain why the item is not applicable/relevant for your study

Not relevant in this study.

11a) If done, who was blinded after assignment to interventions (for example, participants, care providers, those assessing outcomes) and how

NPT: Whether or not administering co-interventions were blinded to group assignment

11a-i) Specify who was blinded, and who wasn't

Specify who was blinded, and who wasn't. Usually, in web-based trials it is not possible to blind the participants [1, 3] (this should be clearly acknowledged), but it may be possible to blind outcome assessors, those doing data analysis or those administering co-interventions (if any).

| | 1 | 2 | 3 | 4 | 5 | |
|---|---|---|---|---|---|---|
| subitem not at all important | ○ | ○ | ○ | ○ | ● | essential |

Effacer la sélection

Does your paper address subitem 11a-i? *

Copy and paste relevant sections from the manuscript (include quotes in quotation marks "like this" to indicate direct quotes from your manuscript), or elaborate on this item by providing additional information not in the ms, or briefly explain why the item is not applicable/relevant for your study

"Two qualified PE teachers, with experience in teaching (10 years) and familiarity with their school's Lü exergaming system, participated in the study. One was the regular PE teacher for the two classes composing EG, and the other for the two classes composing CG. These teachers, as well as the EG and CG students, were informed of the main lines of the study but were left unaware of any expected result."

11a-ii) Discuss e.g., whether participants knew which intervention was the "intervention of interest" and which one was the "comparator"

Informed consent procedures (4a-ii) can create biases and certain expectations - discuss e.g., whether participants knew which intervention was the "intervention of interest" and which one was the "comparator".

| | 1 | 2 | 3 | 4 | 5 | |
|---|---|---|---|---|---|---|
| subitem not at all important | ○ | ○ | ○ | ○ | ● | essential |

Effacer la sélection

Does your paper address subitem 11a-ii?

Copy and paste relevant sections from the manuscript (include quotes in quotation marks "like this" to indicate direct quotes from your manuscript), or elaborate on this item by providing additional information not in the ms, or briefly explain why the item is not applicable/relevant for your study

"Two qualified PE teachers, with experience in teaching (10 years) and familiarity with their school’s Lü exergaming system, participated in the study. One was the regular PE teacher for the two classes composing EG, and the other for the two classes composing CG. These teachers, as well as the EG and CG students, were informed of the main lines of the study but were left unaware of any expected result."

11b) If relevant, description of the similarity of interventions

(this item is usually not relevant for ehealth trials as it refers to similarity of a placebo or sham intervention to a active medication/intervention)

Does your paper address CONSORT subitem 11b? *

Copy and paste relevant sections from the manuscript (include quotes in quotation marks "like this" to indicate direct quotes from your manuscript), or elaborate on this item by providing additional information not in the ms, or briefly explain why the item is not applicable/relevant for your study

Not relevant in this study.

12a) Statistical methods used to compare groups for primary and secondary outcomes

NPT: When applicable, details of whether and how the clustering by care providers or centers was addressed

Does your paper address CONSORT subitem 12a? *

Copy and paste relevant sections from the manuscript (include quotes in quotation marks "like this" to indicate direct quotes from your manuscript), or elaborate on this item by providing additional information not in the ms, or briefly explain why the item is not applicable/relevant for your study

"Data were analyzed using IBM SPSS Statistics, version 30.0.0.0 (171). The normality of the numerical variables was preliminarily checked, and the internal reliability of the situational interest scales was evaluated using Cronbach alpha coefficients.
MANOVA tests were performed to compare the intensity levels of physical activity in EG and CG during sessions 3 and 5 of DEx and TEx. The scores for each intensity level considered were specified as dependent variables and the group condition (EG vs CG) as fixed factor. Similarly, MANOVA tests were done to examine possible differences between EG and CG in scores for each of the three situational interest factors measured after sessions 3 and 5.
For each variable measured at pretest and posttest, ANCOVA tests were performed using a GLM approach [66]. The model included basically the posttest score as the dependent variable, the group (EG vs CG) as fixed factor, and the pretest score as covariate; in the event of between-group difference regarding a situational interest variable, it was planned to add the variable as a covariate. Beyond data normality, variance homogeneity, heteroskedasticity, and regression homogeneity were checked; on such basis, ANCOVA was computed either on untransformed data or on ranks, using the same method for the computations [66,67].
For all the analyses, partial eta squared (ηp2) provided an index of effect size. Contrast analyses were applied in case of significant results."

12a-i) Imputation techniques to deal with attrition / missing values

Imputation techniques to deal with attrition / missing values: Not all participants will use the intervention/comparator as intended and attrition is typically high in ehealth trials. Specify how participants who did not use the application or dropped out from the trial were treated in the statistical analysis (a complete case analysis is strongly discouraged, and simple imputation techniques such as LOCF may also be problematic [4]).

| | 1 | 2 | 3 | 4 | 5 | |
|---|---|---|---|---|---|---|
| subitem not at all important | ○ | ○ | ○ | ○ | ○ | essential |

Does your paper address subitem 12a-i? *

Copy and paste relevant sections from the manuscript (include quotes in quotation marks "like this" to indicate direct quotes from your manuscript), or elaborate on this item by providing additional information not in the ms, or briefly explain why the item is not applicable/relevant for your study

Not relevant in this study.

12b) Methods for additional analyses, such as subgroup analyses and adjusted analyses

Does your paper address CONSORT subitem 12b? *

Copy and paste relevant sections from the manuscript (include quotes in quotation marks "like this" to indicate direct quotes from your manuscript), or elaborate on this item by providing additional information not in the ms, or briefly explain why the item is not applicable/relevant for your study

Not relevant in this study.

X26) REB/IRB Approval and Ethical Considerations [recommended as subheading under "Methods"] (not a CONSORT item)

X26-i) Comment on ethics committee approval

| | 1 | 2 | 3 | 4 | 5 | |
|---|---|---|---|---|---|---|
| subitem not at all important | ○ | ○ | ○ | ○ | ◉ | essential |

Effacer la sélection

Does your paper address subitem X26-i?

Copy and paste relevant sections from the manuscript (include quotes in quotation marks "like this" to indicate direct quotes from your manuscript), or elaborate on this item by providing additional information not in the ms, or briefly explain why the item is not applicable/relevant for your study

"In accordance with local procedure, the project for this pilot interventional study was submitted to the Research Coordination Committee of the host university, based in Switzerland (canton of Vaud). This committee approved the project without requiring further submission to the Cantonal Commission for Ethics in Human Research. Subsequently, this project has been integrated into the university's Research Project Repository.
We also obtained official authorization to implement the study in schools located in the canton of Vaud and selected one secondary school for the study due to its exergaming facilities."

x26-ii) Outline informed consent procedures

Outline informed consent procedures e.g., if consent was obtained offline or online (how? Checkbox, etc.?), and what information was provided (see 4a-ii). See [6] for some items to be included in informed consent documents.

| | 1 | 2 | 3 | 4 | 5 | |
|---|---|---|---|---|---|---|
| subitem not at all important | ○ | ○ | ○ | ◉ | ○ | essential |

Effacer la sélection

Does your paper address subitem X26-ii?

Copy and paste relevant sections from the manuscript (include quotes in quotation marks "like this" to indicate direct quotes from your manuscript), or elaborate on this item by providing additional information not in the ms, or briefly explain why the item is not applicable/relevant for your study

"We presented the project to the school's headteacher, who agreed to its implementation. The teachers involved in the experiment, the parents of the students, and the students completed written informed consent gathered prior to data collection, which gave them the option to withdraw their consent at any time."

X26-iii) Safety and security procedures

Safety and security procedures, incl. privacy considerations, and any steps taken to reduce the likelihood or detection of harm (e.g., education and training, availability of a hotline)

| | 1 | 2 | 3 | 4 | 5 | |
|---|---|---|---|---|---|---|
| subitem not at all important | ○ | ○ | ○ | ◉ | ○ | essential |

Effacer la sélection

Does your paper address subitem X26-iii?

Copy and paste relevant sections from the manuscript (include quotes in quotation marks "like this" to indicate direct quotes from your manuscript), or elaborate on this item by providing additional information not in the ms, or briefly explain why the item is not applicable/relevant for your study

"The data was anonymized and stored on a secure university computer."

RESULTS

13a) For each group, the numbers of participants who were randomly assigned, received intended treatment, and were analysed for the primary outcome

NPT: The number of care providers or centers performing the intervention in each group and the number of patients treated by each care provider in each center

Does your paper address CONSORT subitem 13a? *

Copy and paste relevant sections from the manuscript (include quotes in quotation marks "like this" to indicate direct quotes from your manuscript), or elaborate on this item by providing additional information not in the ms, or briefly explain why the item is not applicable/relevant for your study

Not relevant in this study.

13b) For each group, losses and exclusions after randomisation, together with reasons

Does your paper address CONSORT subitem 13b? (NOTE: Preferably, this is shown in a CONSORT flow diagram) *

Copy and paste relevant sections from the manuscript (include quotes in quotation marks "like this" to indicate direct quotes from your manuscript), or elaborate on this item by providing additional information not in the ms, or briefly explain why the item is not applicable/relevant for your study

Not relevant in this study.

13b-i) Attrition diagram

Strongly recommended: An attrition diagram (e.g., proportion of participants still logging in or using the intervention/comparator in each group plotted over time, similar to a survival curve) or other figures or tables demonstrating usage/dose/engagement.

| | 1 | 2 | 3 | 4 | 5 | |
|---|---|---|---|---|---|---|
| subitem not at all important | ○ | ○ | ○ | ○ | ○ | essential |

Does your paper address subitem 13b-i?

Copy and paste relevant sections from the manuscript or cite the figure number if applicable (include quotes in quotation marks "like this" to indicate direct quotes from your manuscript), or elaborate on this item by providing additional information not in the ms, or briefly explain why the item is not applicable/relevant for your study

Not relevant in this study.

14a) Dates defining the periods of recruitment and follow-up

Does your paper address CONSORT subitem 14a? *

Copy and paste relevant sections from the manuscript (include quotes in quotation marks "like this" to indicate direct quotes from your manuscript), or elaborate on this item by providing additional information not in the ms, or briefly explain why the item is not applicable/relevant for your study

Not relevant in this study.

14a-i) Indicate if critical “secular events” fell into the study period

Indicate if critical “secular events” fell into the study period, e.g., significant changes in Internet resources available or “changes in computer hardware or Internet delivery resources”

| | 1 | 2 | 3 | 4 | 5 | |
|---|---|---|---|---|---|---|
| subitem not at all important | ○ | ○ | ○ | ○ | ○ | essential |

Does your paper address subitem 14a-i?

Copy and paste relevant sections from the manuscript (include quotes in quotation marks "like this" to indicate direct quotes from your manuscript), or elaborate on this item by providing additional information not in the ms, or briefly explain why the item is not applicable/relevant for your study

Not relevant in this study.

14b) Why the trial ended or was stopped (early)

Does your paper address CONSORT subitem 14b? *

Copy and paste relevant sections from the manuscript (include quotes in quotation marks "like this" to indicate direct quotes from your manuscript), or elaborate on this item by providing additional information not in the ms, or briefly explain why the item is not applicable/relevant for your study

The trial did not end / was not stopped before planned.

15) A table showing baseline demographic and clinical characteristics for each group

NPT: When applicable, a description of care providers (case volume, qualification, expertise, etc.) and centers (volume) in each group

Does your paper address CONSORT subitem 15? *

Copy and paste relevant sections from the manuscript (include quotes in quotation marks "like this" to indicate direct quotes from your manuscript), or elaborate on this item by providing additional information not in the ms, or briefly explain why the item is not applicable/relevant for your study

Demographic characteristics of the subjects were given in the Methods/Population section of the manuscript.

15-i) Report demographics associated with digital divide issues

In ehealth trials it is particularly important to report demographics associated with digital divide issues, such as age, education, gender, social-economic status, computer/Internet/ehealth literacy of the participants, if known.

| | 1 | 2 | 3 | 4 | 5 | |
|---|---|---|---|---|---|---|
| subitem not at all important | ○ | ○ | ○ | ○ | ○ | essential |

Does your paper address subitem 15-i? *

Copy and paste relevant sections from the manuscript (include quotes in quotation marks "like this" to indicate direct quotes from your manuscript), or elaborate on this item by providing additional information not in the ms, or briefly explain why the item is not applicable/relevant for your study

Not relevant in this study.

16) For each group, number of participants (denominator) included in each analysis and whether the analysis was by original assigned groups

16-i) Report multiple “denominators” and provide definitions

Report multiple “denominators” and provide definitions: Report N’s (and effect sizes) “across a range of study participation [and use] thresholds” [1], e.g., N exposed, N consented, N used more than x times, N used more than y weeks, N participants “used” the intervention/comparator at specific pre-defined time points of interest (in absolute and relative numbers per group). Always clearly define “use” of the intervention.

| | 1 | 2 | 3 | 4 | 5 | |
|---|---|---|---|---|---|---|
| subitem not at all important | ○ | ○ | ○ | ○ | ○ | essential |

Does your paper address subitem 16-i? *

Copy and paste relevant sections from the manuscript (include quotes in quotation marks "like this" to indicate direct quotes from your manuscript), or elaborate on this item by providing additional information not in the ms, or briefly explain why the item is not applicable/relevant for your study

Not relevant in this study.

16-ii) Primary analysis should be intent-to-treat

Primary analysis should be intent-to-treat, secondary analyses could include comparing only “users”, with the appropriate caveats that this is no longer a randomized sample (see 18-i).

| | 1 | 2 | 3 | 4 | 5 | |
|---|---|---|---|---|---|---|
| subitem not at all important | ○ | ○ | ○ | ○ | ○ | essential |

Does your paper address subitem 16-ii?

Copy and paste relevant sections from the manuscript (include quotes in quotation marks "like this" to indicate direct quotes from your manuscript), or elaborate on this item by providing additional information not in the ms, or briefly explain why the item is not applicable/relevant for your study

Not relevant in this study.

17a) For each primary and secondary outcome, results for each group, and the estimated effect size and its precision (such as 95% confidence interval)

Does your paper address CONSORT subitem 17a? *

Copy and paste relevant sections from the manuscript (include quotes in quotation marks "like this" to indicate direct quotes from your manuscript), or elaborate on this item by providing additional information not in the ms, or briefly explain why the item is not applicable/relevant for your study

For each statistical analysis (MANOVA tests for intensity levels of physical activity, MANOVA tests for situational interest, and ANCOVA tests for mental rotation, general motor coordination, and math achievement), a table has been produced, including F values, P values, and partial eta squared values (Tables 1, 2, and 3).

17a-i) Presentation of process outcomes such as metrics of use and intensity of use

In addition to primary/secondary (clinical) outcomes, the presentation of process outcomes such as metrics of use and intensity of use (dose, exposure) and their operational definitions is critical. This does not only refer to metrics of attrition (13-b) (often a binary variable), but also to more continuous exposure metrics such as “average session length”. These must be accompanied by a technical description how a metric like a “session” is defined (e.g., timeout after idle time) [1] (report under item 6a).

| | 1 | 2 | 3 | 4 | 5 | |
|---|---|---|---|---|---|---|
| subitem not at all important | ○ | ○ | ○ | ○ | ○ | essential |

Does your paper address subitem 17a-i?

Copy and paste relevant sections from the manuscript (include quotes in quotation marks "like this" to indicate direct quotes from your manuscript), or elaborate on this item by providing additional information not in the ms, or briefly explain why the item is not applicable/relevant for your study

Not relevant in this study.

17b) For binary outcomes, presentation of both absolute and relative effect sizes is recommended

Does your paper address CONSORT subitem 17b? *

Copy and paste relevant sections from the manuscript (include quotes in quotation marks "like this" to indicate direct quotes from your manuscript), or elaborate on this item by providing additional information not in the ms, or briefly explain why the item is not applicable/relevant for your study

Not relevant in this study.

18) Results of any other analyses performed, including subgroup analyses and adjusted analyses, distinguishing pre-specified from exploratory

Does your paper address CONSORT subitem 18? *

Copy and paste relevant sections from the manuscript (include quotes in quotation marks "like this" to indicate direct quotes from your manuscript), or elaborate on this item by providing additional information not in the ms, or briefly explain why the item is not applicable/relevant for your study

Not relevant in this study.

18-i) Subgroup analysis of comparing only users

A subgroup analysis of comparing only users is not uncommon in ehealth trials, but if done, it must be stressed that this is a self-selected sample and no longer an unbiased sample from a randomized trial (see 16-iii).

| | 1 | 2 | 3 | 4 | 5 | |
|---|---|---|---|---|---|---|
| subitem not at all important | ○ | ○ | ○ | ○ | ○ | essential |

Does your paper address subitem 18-i?

Copy and paste relevant sections from the manuscript (include quotes in quotation marks "like this" to indicate direct quotes from your manuscript), or elaborate on this item by providing additional information not in the ms, or briefly explain why the item is not applicable/relevant for your study

Not relevant in this study.

19) All important harms or unintended effects in each group
(for specific guidance see CONSORT for harms)

Does your paper address CONSORT subitem 19? *

Copy and paste relevant sections from the manuscript (include quotes in quotation marks "like this" to indicate direct quotes from your manuscript), or elaborate on this item by providing additional information not in the ms, or briefly explain why the item is not applicable/relevant for your study

Not relevant in this study.

19-i) Include privacy breaches, technical problems

Include privacy breaches, technical problems. This does not only include physical “harm” to participants, but also incidents such as perceived or real privacy breaches [1], technical problems, and other unexpected/unintended incidents. “Unintended effects” also includes unintended positive effects [2].

| | 1 | 2 | 3 | 4 | 5 | |
|---|---|---|---|---|---|---|
| subitem not at all important | ○ | ○ | ○ | ○ | ○ | essential |

Does your paper address subitem 19-i?

Copy and paste relevant sections from the manuscript (include quotes in quotation marks "like this" to indicate direct quotes from your manuscript), or elaborate on this item by providing additional information not in the ms, or briefly explain why the item is not applicable/relevant for your study

Not relevant in this study.

19-ii) Include qualitative feedback from participants or observations from staff/researchers

Include qualitative feedback from participants or observations from staff/researchers, if available, on strengths and shortcomings of the application, especially if they point to unintended/unexpected effects or uses. This includes (if available) reasons for why people did or did not use the application as intended by the developers.

| | 1 | 2 | 3 | 4 | 5 | |
|---|---|---|---|---|---|---|
| subitem not at all important | ○ | ○ | ○ | ○ | ○ | essential |

Does your paper address subitem 19-ii?

Copy and paste relevant sections from the manuscript (include quotes in quotation marks "like this" to indicate direct quotes from your manuscript), or elaborate on this item by providing additional information not in the ms, or briefly explain why the item is not applicable/relevant for your study

Not relevant in this study.

DISCUSSION

22) Interpretation consistent with results, balancing benefits and harms, and considering other relevant evidence

NPT: In addition, take into account the choice of the comparator, lack of or partial blinding, and unequal expertise of care providers or centers in each group

22-i) Restate study questions and summarize the answers suggested by the data, starting with primary outcomes and process outcomes (use)

Restate study questions and summarize the answers suggested by the data, starting with primary outcomes and process outcomes (use).

| | 1 | 2 | 3 | 4 | 5 | |
|---|---|---|---|---|---|---|
| subitem not at all important | ○ | ○ | ○ | ○ | ◉ | essential |

Effacer la sélection

Does your paper address subitem 22-i? *

Copy and paste relevant sections from the manuscript (include quotes in quotation marks "like this" to indicate direct quotes from your manuscript), or elaborate on this item by providing additional information not in the ms, or briefly explain why the item is not applicable/relevant for your study

"This pilot interventional study aimed to determine whether dance-based exergaming (DEx), involving mental rotation to perform different types of locomotion and interlimb coordination, may influence: (1) mental rotation efficiency, (2) general motor coordination, and (3) math achievement in an experimental group (EG) of adolescent students. This was done using a pretest-posttest design, including a control group (CG) involved in exergaming based on precision ball-throwing (TEx) and monitoring physical activity intensity levels and situational interest.
Non-instrumented observation suggested that the sessions proceeded as planned. This was corroborated by physical activity monitoring (Table 1) [...]. This suggested that situational interest did not impede the physical activity required by DEx and TEx. Only the scores for the triggered situational interest factor in session 5 significantly differed between groups, with a higher score in EG than in CG (Table 2). ANCOVAs were performed controlling for this variable; the results are discussed below."
"DEx was likely to elicit mental rotation training in EG, whereas mental rotation was not relevant during TEx in CG. The advantage for EG over CG regarding mental rotation found, once DEx and TEx were completed, may thus be due to mental rotation training in EG during DEx."
"Thus, DEx and TEx showed similar influence on the performance of the other parts of the circuit, and the performance improved on average from 22.41 (SD 4.15) to 21.72 (SD 4.39) seconds in EG (3% improvement) and from 22.42 (SD 3.44) to 21.20 (SD 3.41) seconds in CG (5% improvement)."
"Our results confirmed such influence of dance-based exergaming when compared with exergaming based on precision ball-throwing. However, this influence was found limited to peculiar mathematical tasks (Table 3). [...] According to the literature [72], this advantage for EG might be limited to tasks primarily requiring procedural strategies for calculating, rather than strategies for retrieving memorized results."

22-ii) Highlight unanswered new questions, suggest future research

Highlight unanswered new questions, suggest future research.

| | 1 | 2 | 3 | 4 | 5 | |
|---|---|---|---|---|---|---|
| subitem not at all important | ○ | ○ | ○ | ● | ○ | essential |

Effacer la sélection

Does your paper address subitem 22-ii?

Copy and paste relevant sections from the manuscript (include quotes in quotation marks "like this" to indicate direct quotes from your manuscript), or elaborate on this item by providing additional information not in the ms, or briefly explain why the item is not applicable/relevant for your study

"The results of this study encourage to clarify whether mental rotation training through exergaming may influence math achievement. These results also encourage [...] to determine if the influence of exergaming on the performance of arithmetical tasks is limited to tasks requiring procedural strategies for calculation. This also raises the question of whether such influence might depend on age, gender, or math experience. [...] Moreover, this study does not allow conclusions on exergaming influence on general motor coordination; the results encourage further research to clarify this influence and its potential links with mental rotation training during exergaming. It would also be of interest to assess the potential added value of specific exergaming features (e.g., avatar demonstration, or options for managing exercise variety and progression) in achieving the effects observed in our study. Teacher interventions directly linked to such features could complementarily be considered. This assessment might be done by comparison with a conventional physical activity."

20) Trial limitations, addressing sources of potential bias, imprecision, and, if relevant, multiplicity of analyses

20-i) Typical limitations in ehealth trials

Typical limitations in ehealth trials: Participants in ehealth trials are rarely blinded. Ehealth trials often look at a multiplicity of outcomes, increasing risk for a Type I error. Discuss biases due to non-use of the intervention/usability issues, biases through informed consent procedures, unexpected events.

| | 1 | 2 | 3 | 4 | 5 | |
|---|---|---|---|---|---|---|
| subitem not at all important | ○ | ○ | ○ | ○ | ◉ | essential |

Effacer la sélection

Does your paper address subitem 20-i? *

Copy and paste relevant sections from the manuscript (include quotes in quotation marks "like this" to indicate direct quotes from your manuscript), or elaborate on this item by providing additional information not in the ms, or briefly explain why the item is not applicable/relevant for your study

"One limitation is linked to the implementation of scientific research in a school environment. Notably, this did not allow us to allocate students randomly between EG and CG. Possible generalization of the results should also be questioned, especially as these results might depend on features of the exergaming sequences implemented, of the school of implementation, and of the students involved in the sequences. Confirmation of our results could also be sought using larger samples and longer sequences than in this study."

21) Generalisability (external validity, applicability) of the trial findings

NPT: External validity of the trial findings according to the intervention, comparators, patients, and care providers or centers involved in the trial

### 21-i) Generalizability to other populations

Generalizability to other populations: In particular, discuss generalizability to a general Internet population, outside of a RCT setting, and general patient population, including applicability of the study results for other organizations

| | 1 | 2 | 3 | 4 | 5 | |
|---|---|---|---|---|---|---|
| subitem not at all important | ○ | ○ | ○ | ◉ | ○ | essential |

Effacer la sélection

### Does your paper address subitem 21-i?

Copy and paste relevant sections from the manuscript (include quotes in quotation marks "like this" to indicate direct quotes from your manuscript), or elaborate on this item by providing additional information not in the ms, or briefly explain why the item is not applicable/relevant for your study

"Possible generalization of the results should also be questioned, especially as these results might depend on features of the exergaming sequences implemented, of the school of implementation, and of the students involved in the sequences. Confirmation of our results could also be sought using larger samples and longer sequences than in this study."

### 21-ii) Discuss if there were elements in the RCT that would be different in a routine application setting

Discuss if there were elements in the RCT that would be different in a routine application setting (e.g., prompts/reminders, more human involvement, training sessions or other co-interventions) and what impact the omission of these elements could have on use, adoption, or outcomes if the intervention is applied outside of a RCT setting.

| | 1 | 2 | 3 | 4 | 5 | |
|---|---|---|---|---|---|---|
| subitem not at all important | ○ | ○ | ○ | ○ | ○ | essential |

Does your paper address subitem 21-ii?

Copy and paste relevant sections from the manuscript (include quotes in quotation marks "like this" to indicate direct quotes from your manuscript), or elaborate on this item by providing additional information not in the ms, or briefly explain why the item is not applicable/relevant for your study

Not considered in this study.

OTHER INFORMATION

23) Registration number and name of trial registry

Does your paper address CONSORT subitem 23? *

Copy and paste relevant sections from the manuscript (include quotes in quotation marks "like this" to indicate direct quotes from your manuscript), or elaborate on this item by providing additional information not in the ms, or briefly explain why the item is not applicable/relevant for your study

Not relevant in this study.

24) Where the full trial protocol can be accessed, if available

Does your paper address CONSORT subitem 24? *

Cite a Multimedia Appendix, other reference, or copy and paste relevant sections from the manuscript (include quotes in quotation marks "like this" to indicate direct quotes from your manuscript), or elaborate on this item by providing additional information not in the ms, or briefly explain why the item is not applicable/relevant for your study

Not relevant in this study.

25) Sources of funding and other support (such as supply of drugs), role of funders

Does your paper address CONSORT subitem 25? *

Copy and paste relevant sections from the manuscript (include quotes in quotation marks "like this" to indicate direct quotes from your manuscript), or elaborate on this item by providing additional information not in the ms, or briefly explain why the item is not applicable/relevant for your study

"This work was supported by the Reserve and Innovation Fund of the University of Teacher Education (HEP-VD), 1007 Lausanne, Switzerland (grant number 10_FRI_A21_Fargier)."

X27) Conflicts of Interest (not a CONSORT item)

X27-i) State the relation of the study team towards the system being evaluated

In addition to the usual declaration of interests (financial or otherwise), also state the relation of the study team towards the system being evaluated, i.e., state if the authors/evaluators are distinct from or identical with the developers/sponsors of the intervention.

| | 1 | 2 | 3 | 4 | 5 | |
|---|---|---|---|---|---|---|
| subitem not at all important | ○ | ○ | ○ | ○ | ◉ | essential |

Effacer la sélection

Does your paper address subitem X27-i?

Copy and paste relevant sections from the manuscript (include quotes in quotation marks "like this" to indicate direct quotes from your manuscript), or elaborate on this item by providing additional information not in the ms, or briefly explain why the item is not applicable/relevant for your study

Not relevant in this study (simple use of the system).

About the CONSORT EHEALTH checklist

As a result of using this checklist, did you make changes in your manuscript? *

- ○ yes, major changes
- ◉ yes, minor changes
- ○ no

What were the most important changes you made as a result of using this checklist?

Ethical considerations and teacher involvement in the study.

How much time did you spend on going through the checklist INCLUDING making changes in your manuscript *

30 hours were spent reviewing the checklist and re-examining the manuscript.

As a result of using this checklist, do you think your manuscript has improved? *

- ◉ yes
- ○ no
- ○ Autre :

Would you like to become involved in the CONSORT EHEALTH group?

This would involve for example becoming involved in participating in a workshop and writing an "Explanation and Elaboration" document

- ( ) yes
- (•) no
- ( ) Autre :

Effacer la sélection

Any other comments or questions on CONSORT EHEALTH

Votre réponse

STOP - Save this form as PDF before you click submit

To generate a record that you filled in this form, we recommend to generate a PDF of this page (on a Mac, simply select "print" and then select "print as PDF") before you submit it.

When you submit your (revised) paper to JMIR, please upload the PDF as supplementary file.

Don't worry if some text in the textboxes is cut off, as we still have the complete information in our database. Thank you!

Final step: Click submit !

Click submit so we have your answers in our database!

Envoyer Effacer le formulaire

N'envoyez jamais de mots de passe via Google Forms.

Ce contenu n'est ni rédigé, ni cautionné par Google. - Contacter le propriétaire du formulaire - Conditions d'utilisation - Règles de confidentialité

Ce formulaire vous semble suspect ? Signaler

Google Forms
